# Supplementary material for: An epidemiological study of season of birth, mental health, and neuroimaging in the UK Biobank
Source: PLoS One. 2024 May 22;19(5):e0300449. doi: 10.1371/journal.pone.0300449 (PMC11111058; doi:10.1371/journal.pone.0300449)
Supplement: S1 File — (DOCX) [file pone.0300449.s001.docx]

**Supplementary Information**

**Table of Contents**

Supplementary Methods

S1. UKB Data procedures and acquisition

- S1.1 Brain imaging measures

S2. Data pre-processing

S2.1 Covariates

- - - S2.1.1 Mental health traits
  - S2.1.2 Brain imaging measures

S2.2 Classification

- S2.2.1 Mental health traits
  - S2.2.1A Variables utilised to derive probable mania mental health phenotypes
  - S2.2.1B Variables utilised to derive probable depression mental health phenotypes
  - S2.2.1C Criteria for mental health trait phenotype grouping
  - S2.2.1D Overlaps in mental health phenotype groupings
- S2.2.2 Brain imaging measures
  - S2.2.2A UKB T1 and DTI brain imaging variables used to derive brain imaging measures
  - S2.2.2B Variance explained by the first principal component for DTI PCA

S2.3 Quality control

- - S2.3.1 Mental health traits
    - - S2.3.1A Conditions excluded for mental health traits
      - S2.3.1B Sample size per mental health traits post-exclusions

S2.4 Multiple testing correction

- - - - S2.4.1 Multiple testing corrections applied per neuroimaging measure modality

S2.5 Secondary phenotype demographic tables for brain imaging measure associations

- - - - S2.5.1 Demographic table for Probable Major Depressive Disorder secondary phenotype
      - S2.5.2 Demographic table for summer and winter born Probable Major Depressive Disorder secondary phenotype
      - S2.5.3 Demographic table Probable Recurrent Major Depressive Disorder and Probable Single episode Major Depressive disorder secondary phenotype

Supplementary Results

S3. Brain imaging measures supplementary results

- S3.1 Seasonality associations with brain imaging measures
  - S3.1.1 Global T1 measures
  - S3.1.2 Lobar T1 measures
  - S3.1.3 Individual T1 measures
  - S3.1.4 Subcortical Measures
  - S3.1.5 DTI Global Measures
  - S3.1.6 DTI Grouped Tract Measures
  - S3.1.7 DTI Individual Tract Measures
- S3.2 Seasonality associations with brain imaging measures covarying for birth weight
  - S3.2.1 Global T1 measures
  - S3.2.2 Lobar T1 measures
  - S3.2.3 Individual T1 measures
  - S3.2.4 Subcortical Measures
  - S3.2.5 DTI Global Measures
  - S3.2.6 DTI Grouped Tract Measures
  - S3.2.7 DTI Individual Tract Measures
- S3.3 Seasonality associations with mental health traits
- S3.4 Mental health trait associations with brain imaging measures
  - S3.4.1 Global T1 measures
  - S3.4.2 Lobar T1 measures
  - S3.4.3 Individual T1 measures
  - S3.4.4 Subcortical Measures
  - S3.4.5 DTI Global Measures
  - S3.4.6 DTI Grouped Tract Measures
  - S3.4.7 DTI Individual Tract Measures
- S3.5 Secondary phenotype associations with brain imaging measures
  - S3.5.1 Global T1 measures
  - S3.5.2 Lobar T1 measures
  - S3.5.3 Individual T1 measures
  - S3.5.4 Subcortical Measures
  - S3.5.5 DTI Global Measures
  - S3.5.6 DTI Grouped Tract Measures
  - S3.5.7 DTI Individual Tract Measures
- S3.6 Mental health trait and seasonality interactions with brain imaging measures
  - S3.6.1 Global T1 measures
  - S3.6.2 Lobar T1 measures
  - S3.6.3 Individual T1 measures
  - S3.6.4 Subcortical Measures
  - S3.6.5 DTI Global Measures
  - S3.6.6 DTI Grouped Tract Measures
  - S3.6.7 DTI Individual Tract Measures
- S3.7 Seasonality associations with brain imaging measures when covarying for mental health traits and the interaction between mental health traits and seasonality
  - S3.7.1 Global T1 measures
  - S3.7.2 Lobar T1 measures
  - S3.7.3 Individual T1 measures
  - S3.7.4 Subcortical Measures
  - S3.7.5 DTI Global Measures
  - S3.7.6 DTI Grouped Tract Measures
  - S3.7.7 DTI Individual Tract Measures

S4. Summary of directions of effect for variables with significant associations for all analyses
S5. Extended results of mental health trait associations with brain imaging measures

**SUPPLEMENTARY METHODS**

**S1. UKB Data procedures and acquisition**

***S1.1 Brain Imaging Measures***

Participants were scanned over three locations (Cheadle, Newcastle and Reading) using a Siemens Skyra 3T scanner with a standard Siemens 32-channel RF receive head coil. For this study, T1 and DTI brain imaging measures were extracted from UKB after undergoing a standard pre-processing pipeline [1]. Full protocol and acquisition parameters are available at <https://biobank.ctsu.ox.ac.uk/crystal/crystal/docs/brain_mri.pdf> and <https://www.fmrib.ox.ac.uk/ukbiobank/>.

T1 measures were further processed by UKB with Freesurfer 6.0 software whereby IDPs are extracted in reference to standard surface area, volume and mean cortical thickness given by standard atlases, in this case the Desikan-Killiany-Tourville atlas was utilised [2]. For subcortical regions Freesurfer ASEG was used [3,4]. All output is then subjected to Qoala-T QC checks, with any output close to the threshold also being manually checked [5].

DTI measures are additionally corrected for head motion and eddy currents and fitted with the DTIFIT tool to create fractional anisotropy (FA) and mean diffusivity (MD) outputs. For this study, three tracts were not included in any FA or MD tract group measures (corpus callosum, corona radiata and internal capsule).

1. Alfaro-Almagro F, Jenkinson M, Bangerter NK, Andersson JLR, Griffanti L, Douaud G, et al. Image processing and Quality Control for the first 10,000 brain imaging datasets from UK Biobank. NeuroImage. 2018;166: 400–424. doi:10.1016/j.neuroimage.2017.10.034

2. Alexander B, Loh WY, Matthews LG, Murray AL, Adamson C, Beare R, et al. Desikan-Killiany-Tourville Atlas Compatible Version of M-CRIB Neonatal Parcellated Whole Brain Atlas: The M-CRIB 2.0. Front Neurosci. 2019;13: 34. doi:10.3389/fnins.2019.00034

3. Fischl B, Salat DH, Busa E, Albert M, Dieterich M, Haselgrove C, et al. Whole brain segmentation: automated labeling of neuroanatomical structures in the human brain. Neuron. 2002;33: 341–355. doi:10.1016/s0896-6273(02)00569-x

4. Iglesias JE, Augustinack JC, Nguyen K, Player CM, Player A, Wright M, et al. A computational atlas of the hippocampal formation using ex vivo, ultra-high resolution MRI: Application to adaptive segmentation of in vivo MRI. NeuroImage. 2015;115: 117–137. doi:10.1016/j.neuroimage.2015.04.042

5. Klapwijk ET, van de Kamp F, van der Meulen M, Peters S, Wierenga LM. Qoala-T: A supervised-learning tool for quality control of FreeSurfer segmented MRI data. NeuroImage. 2019;189: 116–129. doi:10.1016/j.neuroimage.2019.01.014

**S2. Data pre-processing**

***S2.1 Covariates***

***S2.1.1 Mental health traits***

Variables and UKB data-fields used as covariates used for mental health trait regression models.

| **Covariate** | **UKB data-field** |
| --- | --- |
| Sex | 31 |
| Age | 21003 |
| Age^2^ | - |
| Assessment centre | 54 |
| Townsend Deprivation Index | 189 |
| Birth Location | 129, 130* |

******* Place of birth co-ordinates were collected by UKB as Ordnance Survey grid references <https://biobank.ndph.ox.ac.uk/showcase/showcase/docs/UKgrid.pdf> referring to easting and northing with a reference point close to the Isles of Sicily. Since they adequately tracked north and east directions within the UK in relation to its geography and to each other, they were not converted to true longitude and latitude for this study. Instead, a kmeans clustering approach was utilised whereby participants were clustered by UKB datafields 129 and 130 to derive a birth location cluster after scaling these measures. A maximum of 12 clusters were inspected and the optimal number of clusters was chosen by visual inspection of an elbow chart (See Figures 1-3 below).


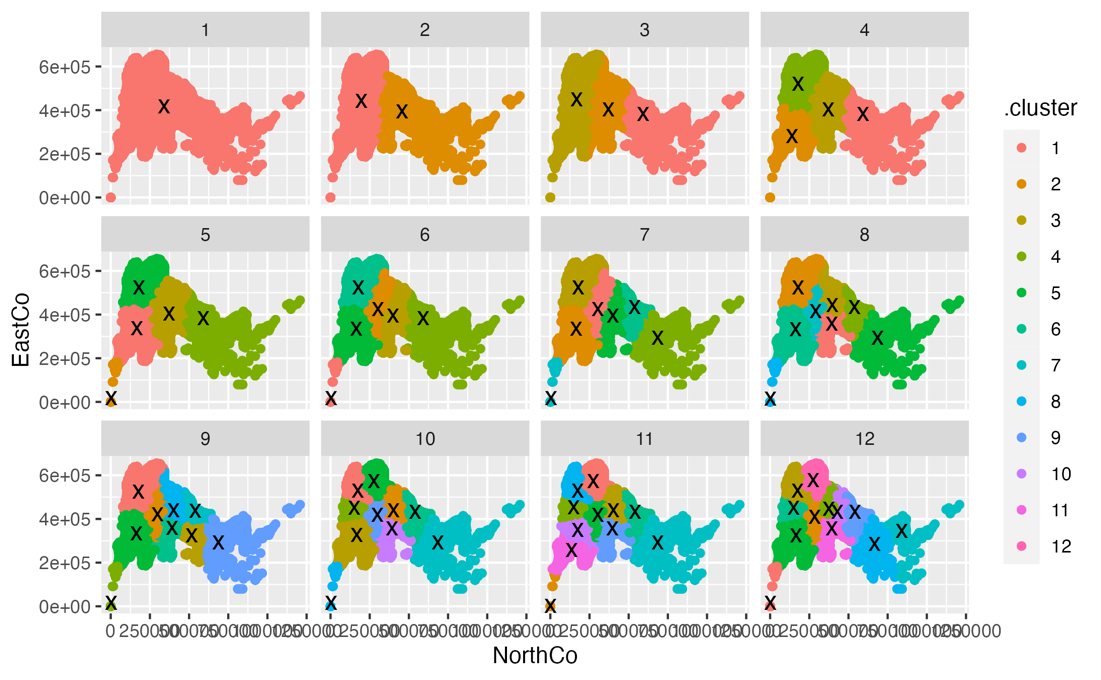


**S1 Fig.** Visualisation of a maximum of 12 clusters for birth location via k-means clustering analysis on UKB datafields 129 and 130 for participants who had completed the MHQ and/or mania and depression questions in the touchscreen questionnaire.


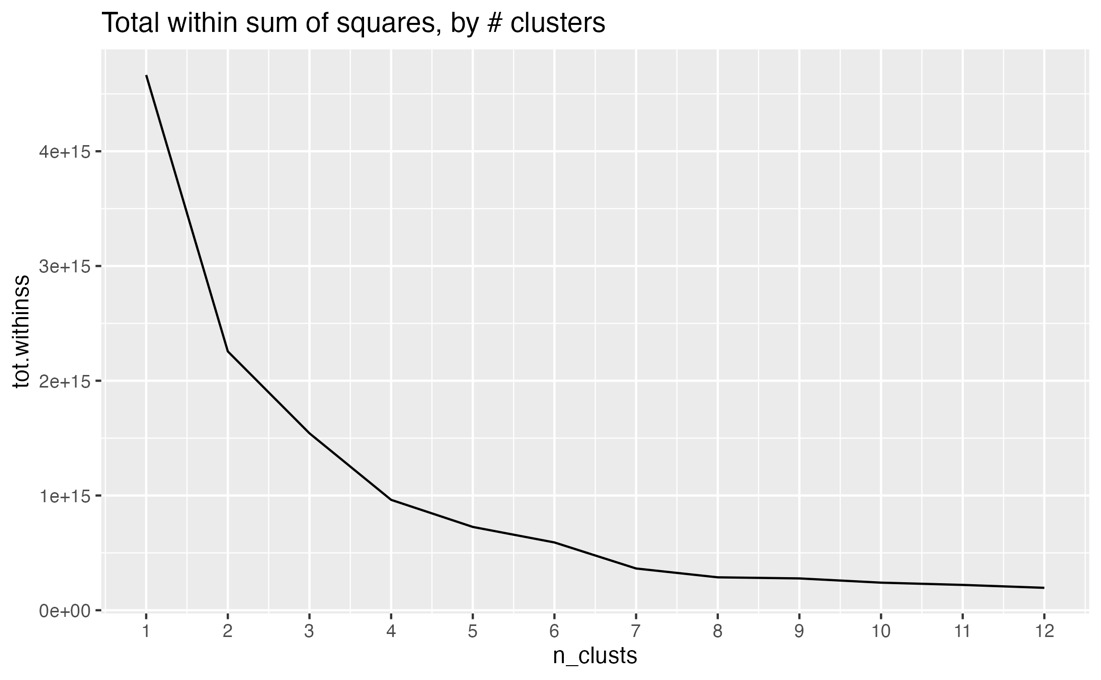


**S2 Fig.** Elbow chart of the total within sum of squares by number of clusters (max N= 12) for kmeans clustering performed on UKB datafields 129 and 130 for participants who had completed the MHQ and/or mania and depression questions in the touchscreen questionnaire.


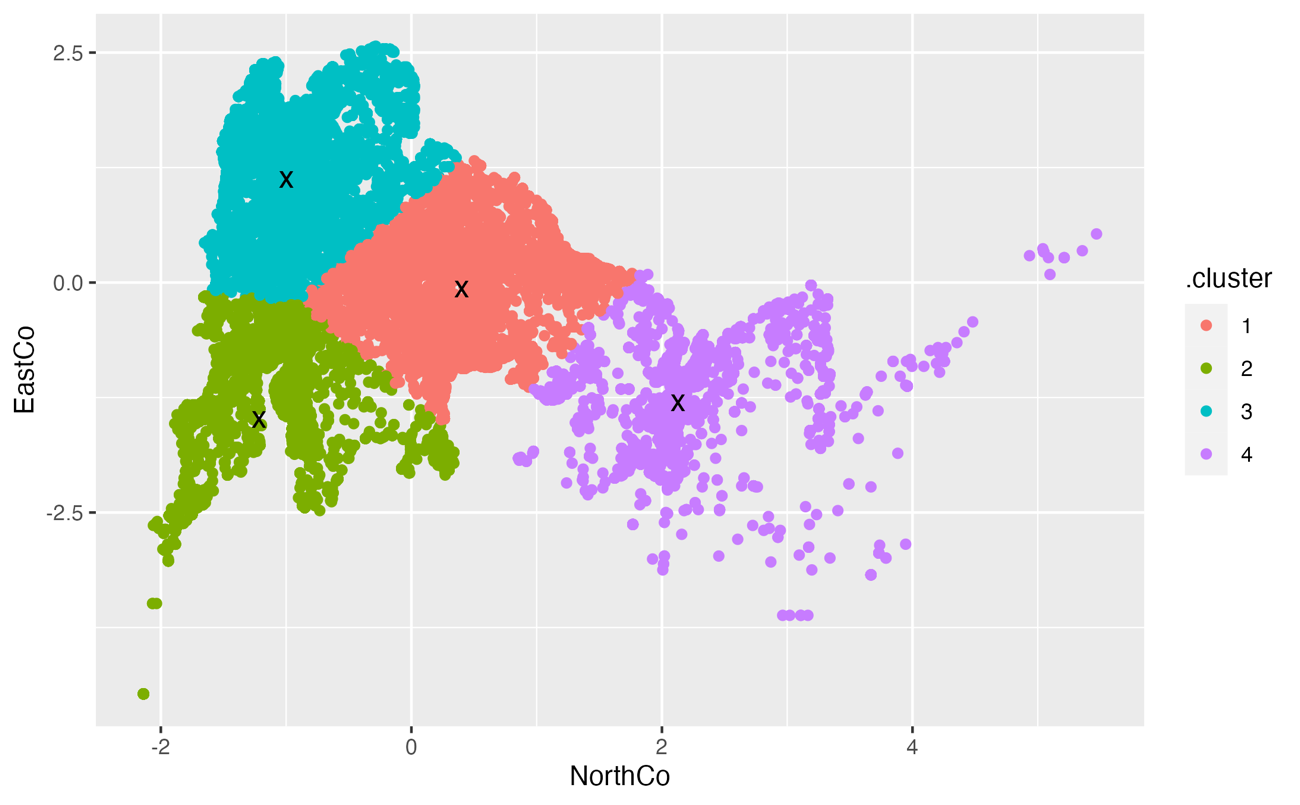


**S3 Fig.** Visualisation of the final four clusters chosen to be the proxy for birth location via k-means clustering analysis on UKB datafields 129 and 130 for participants who had completed the MHQ and/or mania and depression questions in the touchscreen questionnaire.

***S2.1.2 Brain imaging measures***

Variables and UKB data-fields used as covariates used for brain imaging measure regression models.

| **Covariate** | **UKB data-field** |
| --- | --- |
| ***Partially adjusted*** |  |
| Sex | 31 |
| Age | 21003 |
| Age^2^ | - |
| Assessment centre | 54 |
| Standardised intracranial volume | Sum of: 25005, 25007, 25003 |
| Scanner lateral (X) brain position | 25756 |
| Scanner transverse (Y) brain position | 25757 |
| Scanner longitudinal (Z) brain position | 25758 |
| Scanner table position | 25759 |
| Townsend Deprivation Index | 189 |
| Birth Location | 129, 130* |
| ***Maximally adjusted - all of the above plus:*** |  |
| Birth Weight | 20022 |

******* Place of birth co-ordinates were collected by UKB as Ordnance Survey grid references <https://biobank.ndph.ox.ac.uk/showcase/showcase/docs/UKgrid.pdf> referring to easting and northing with a reference point close to the Isles of Sicily. Since they adequately tracked north and east directions within the UK in relation to its geography and to each other, they were not converted to true longitude and latitude for this study. Instead, a kmeans clustering approach was utilised whereby participants were clustered by UKB datafields 129 and 130 to derive a birth location cluster after scaling these measures. A maximum of 12 clusters were inspected and the optimal number of clusters was chosen by visual inspection of an elbow chart (See Figures 4-6 below).


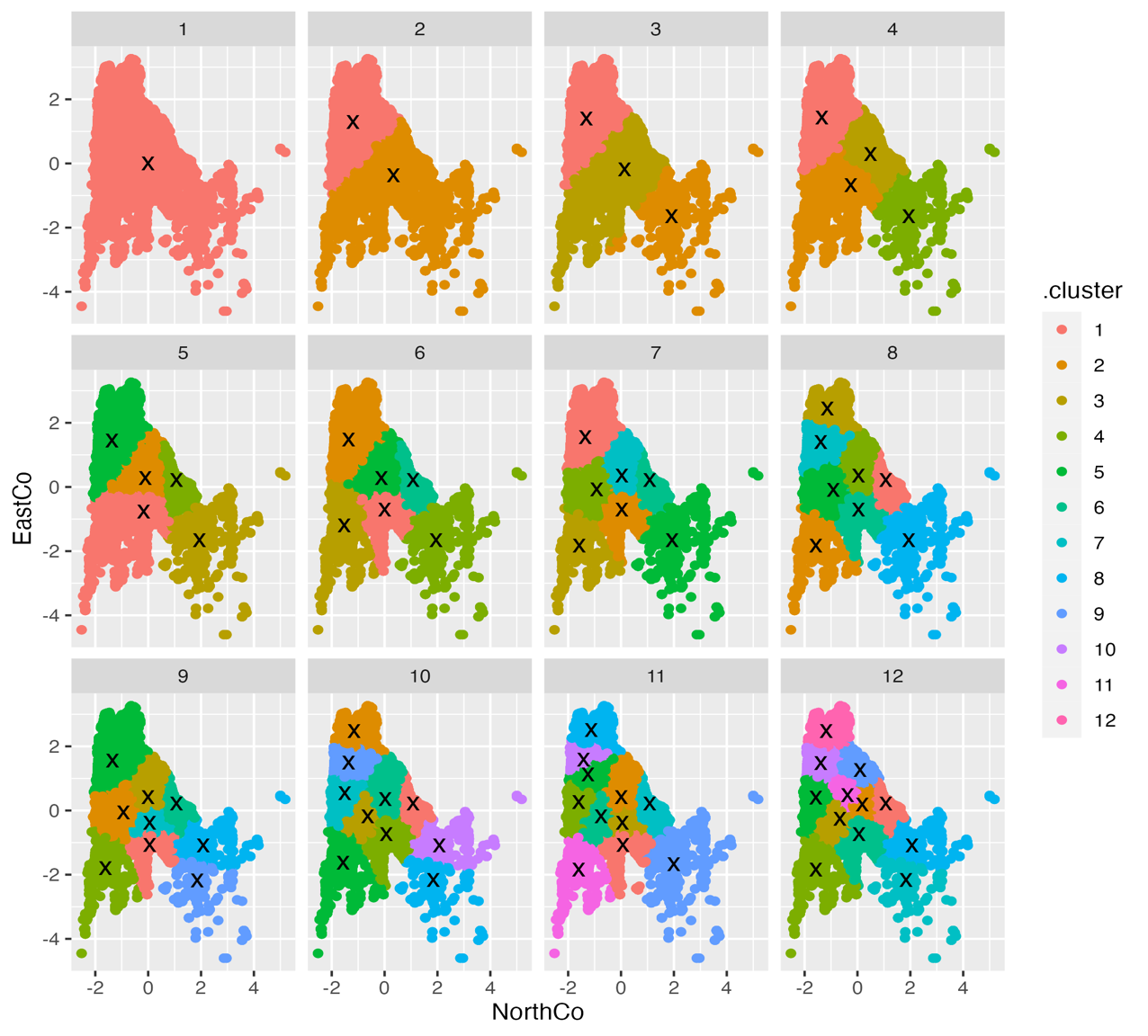


**S4 Fig.** Visualisation of a maximum of 12 clusters for birth location via k-means clustering analysis on UKB datafields 129 and 130 for participants who had attended UKB’s imaging assessment.


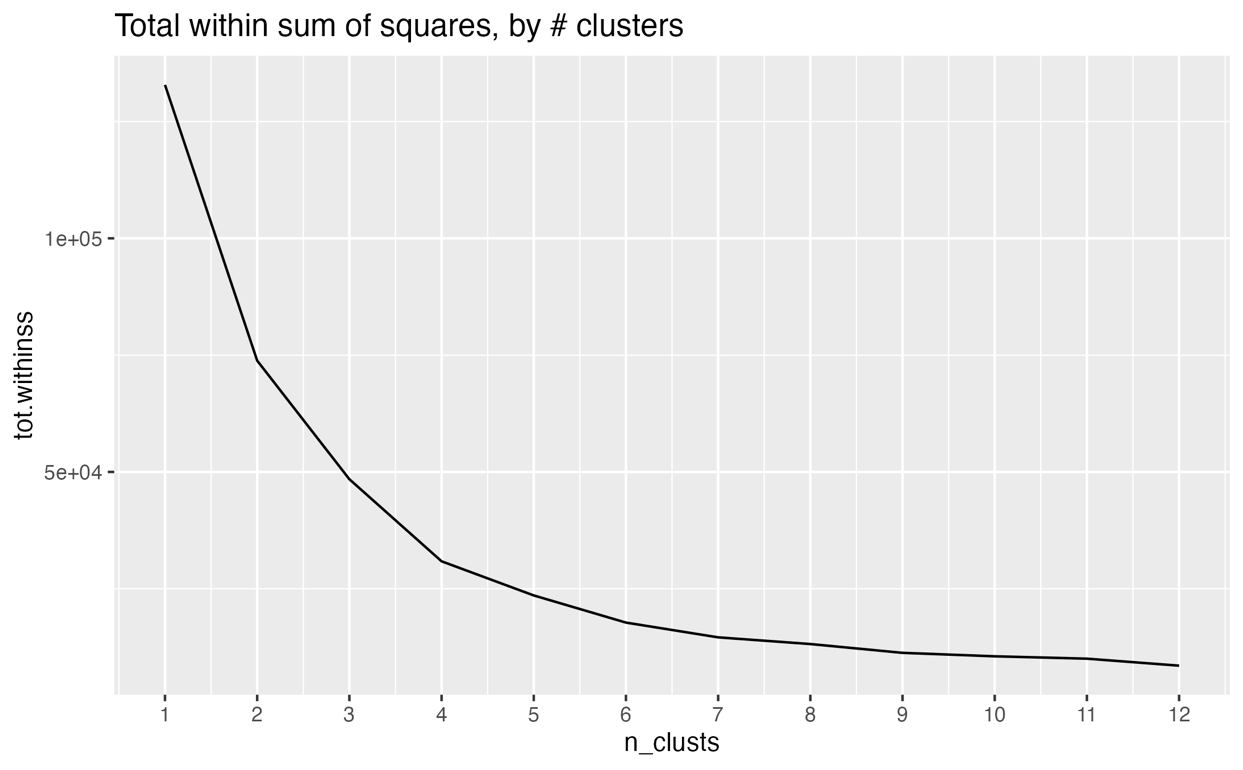


**S5 Fig.** Elbow chart of the total within sum of squares by number of clusters (max N= 12) for kmeans clustering performed on UKB datafields 129 and 130 for participants who had attended UKB’s imaging assessment.


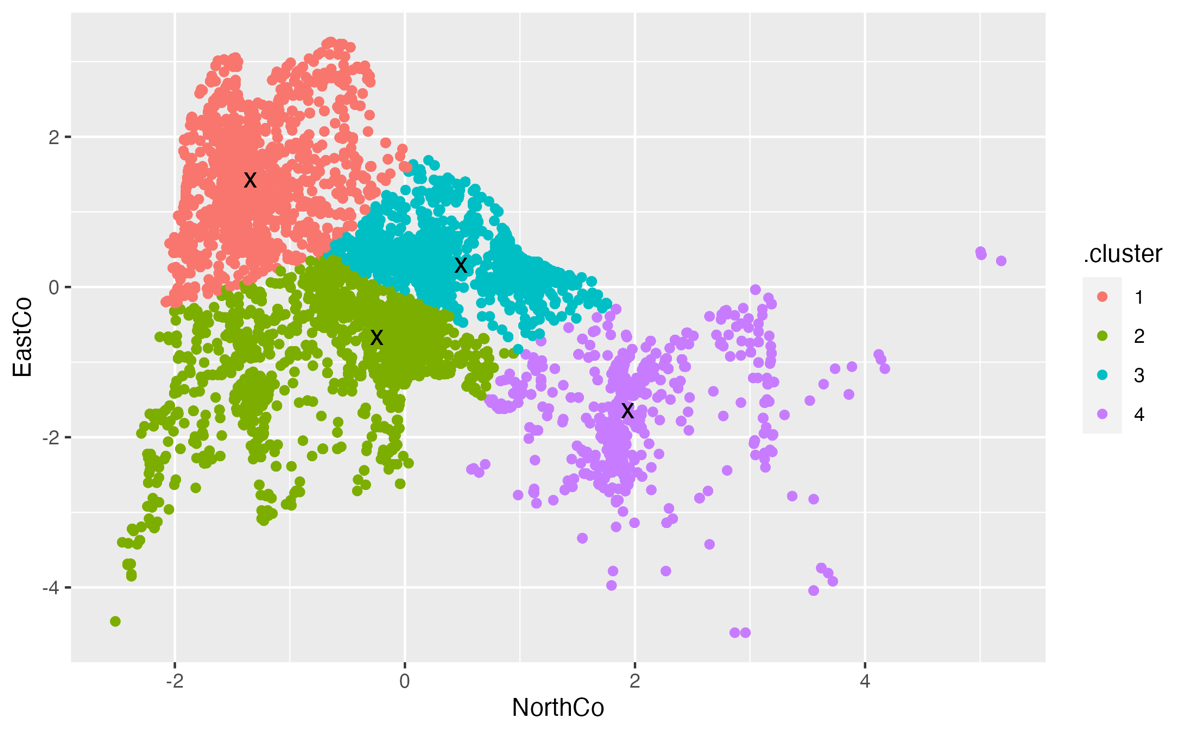


**S6 Fig.** Visualisation of the final four clusters chosen to be the proxy for birth location via k-means clustering analysis on UKB datafields 129 and 130 for participants who had attended UKB’s imaging assessment.

***S2.2 Classification***

***S2.2.1 Mental health traits***

***S2.2.1A Variables utilised to derive probable mental health phenotypes***

UBK variables used to derive probable mania, probable hypomania and no mania (control) mental health trait phenotypes from the Thoughts and Feelings Questionnaire and Touchscreen Mental Health Questionnaire.

|  | **Thoughts and Feelings Questionnaire (MHQ)** | **UKB Data-Fields** | **Touchscreen Mental Health Questionnaire** | **UKB Data-Fields** |
| --- | --- | --- | --- | --- |
| ***Probable Mania*** | Yes to hyper/manic for two days  OR | 20501 | Yes to hyper/manic for two days  OR | 4642 |
|  | Yes to irritable/argumentative for two days | 20502 | Yes to irritable/argumentative for two days | 4653 |
|  | Three or more symptoms | 20548 | Three or more symptoms | 6156 |
|  | Episode length of one week or more | 20492 | Episode length of one week or more | 5663 |
|  | Symptoms affected daily activities | 20493 | Symptoms affected daily activities | 5674 |
| ***Probable Hypomania*** | Yes to hyper/manic for two days  OR | 20501 | Yes to hyper/manic for two days  OR | 4642 |
|  | Yes to irritable/argumentative for two days | 20502 | Yes to irritable/argumentative for two days | 4653 |
|  | Three or more symptoms | 20548 | Three or more symptoms | 6156 |
|  | Episode length of 24 hours or more | 20492 | Episode length of two days or more | 5663 |
| ***No Mania*** | No to hyper/manic for two days  AND | 20501 | No to hyper/manic for two days  AND | 4642 |
|  | No to irritable/argumentative for two days | 20502 | No to irritable/argumentative for two days | 4653 |

***S2.2.1B Variables utilised to derive probable depression mental health phenotypes***

UKB variables utilised to derive probable singular episode of major depression, probable recurrent depression and no probable depression (control) mental health trait phenotypes from the Thoughts and Feelings Questionnaire and Touchscreen Mental Health Questionnaire.

| **Phenotype** | **Thoughts and Feelings Questionnaire (MHQ)** | **UKB Data-Fields** | **Touchscreen Mental Health Questionnaire** | **UKB Data-Fields** |
| --- | --- | --- | --- | --- |
| ***Probable Single Episode of Major Depression*** | Yes to depressed feelings for two+ weeks  OR | 20446 | Yes to depressed feelings for one week  OR | 4598 |
|  | Yes to anhedonia for two+ weeks | 20441 | Yes to anhedonia for one week | 4631 |
|  | Five or more symptoms | 20446, 20441, 20536, 20532, 20450, 20435, 20437 | Maximum period of depression/anhedonia lasted two+ weeks | 4609 |
|  | One episode over lifetime | 20442 | One episode over lifetime | 4620 |
|  | Professional informed about depression | 20448 | GP or psychiatrist seen for nerves/anxiety/tension/depression | 2090, 2100 |
| ***Probable Recurrent Depression*** | Yes to depressed feelings for two+ weeks  OR | 20446 | Yes to depressed feelings for one week  OR | 4598 |
|  | Yes to anhedonia for two+ weeks | 20441 | Yes to anhedonia for one week | 4631 |
|  | Five or more symptoms | 20446, 20441, 20536, 20532, 20450, 20435, 20437 | Maximum period of depression/anhedonia lasted two+ weeks | 4609 |
|  | More than one episode over lifetime | 20442 | More than one episode over lifetime | 4620 |
|  | Professional informed about depression | 20448 | GP or psychiatrist seen for nerves/anxiety/tension/depression | 2090, 2100 |
| ***No Probable Depression*** | No to depressed feelings for two+ weeks AND | 20446 | No to depressed feelings for one week AND | 4598 |
|  | No to anhedonia for two+ weeks | 20441 | No to anhedonia for one week | 4631 |

***S2.2.1C Criteria for mental health trait phenotype groupings***

Criteria used to define final mental health trait phenotype groupings based on participant response type to questions from UKB Thoughts and Feelings Questionnaire and UKB Touchscreen Mental Health Questionnaire.

******

***S2.2.1D Overlaps in mental health phenotype groupings***

Overlaps permitted within the final mental health phenotype groupings. All participants in the control group had answered ‘no’ to leading probable mania and/or probable depression.

|  | **Probable Unipolar Mania** |  |  |  | **Probable Bipolar Depression** |  | **Probable Recurring MDD** | **Probable Single Episode MDD** | **Control Group** |
| --- | --- | --- | --- | --- | --- | --- | --- | --- | --- |
| **Probable Mania** | Y | N | Y | Y | N | N | N | N | N |
| **Probable Hypomania** | N | Y | N | N | Y | Y | N | N | N |
| **Probable Recurring MDD** | N | N | Y | N | Y | N | Y | N | N |
| **Probable Single Episode MDD** | N | N | N | Y | N | Y | N | Y | N |

***S2.2.2 Brain imaging measures***

***S2.2.2A UKB T1 and DTI brain imaging variables used to derive brain imaging measures***

UKB variables used to derive brain imaging measures per participant. Individual T1 measures per hemisphere were extracted for each participant from the Freesurfer DKT category (196) for bilateral measures and unilaterally for unilateral measures. Lobar and global T1 measures were composed per participant as below. Subcortical structures were extracted from the UKB ‘FIRST’ category (1102) by hemisphere. FA and MD DTI measures were extracted per hemisphere for bilateral structures from the ‘dMRI weighted means’ UKB category (135) and unilaterally for unilateral measures.

| **Variable** | **Measures** |
| --- | --- |
| ***T1 Global Measures*** |  |
| Global Cortical Volume | Sum of 5 lobar measures |
| Global Cortical Thickness | Weighted average of thickness of the 5 lobes multiplied by their surface area and divided by their thickness. |
| Global Surface Area | Sum of 5 lobar measures |
| ***T1 Lobes*** |  |
| Frontal | Sum of: Superior frontal gyrus, Rostral middle frontal, caudal middle frontal, Pars orbitalis, pars triangularis, pars opercularis, Lateral orbitofrontal, medial orbitofrontal, Precentral gyrus, Paracentral cortex |
| Temporal | Sum of: Insula, Superior temporal, transverse temporal, Middle temporal gyrus, Inferior temporal gyrus, Fusiform, parahippocampal, entorhinal |
| Parietal | Sum of: Postcentral gyrus, paracentral cortex, Superior parietal cortex, Inferior parietal cortex, Supramarginal gyrus, Precuneus |
| Occipital | Sum of: Lateral occipital cortex, Cuneus, Pericalcarine cortex, Lingual gyrus |
| Cingulate | Sum of: Rostral anterior cingulate cortex, Caudal anterior cingulate cortex, Posterior cingulate cortex, Cingulate isthmus |
| ***T1 Individual Structures*** |  |
| 31 Cortical Regions | All of the above in the lobe categories |
| 7 Subcortical | Nucelus accumbens, amygdala, caudate nucleus, hippocampus, pallidum, putamen and thalamus |
| ***DTI Measures*** |  |
| gFA/MD | PCA of all tracts |
| gAssociation Fibres FA/MD | PCA of 6 bilateral tracts; Cingulum-Cingulate Gyrus, Parahippocampal part of cingulum, inferior fronto-occipital-fasciculus, superior longitudinal fasciculus, uncinate fasciculus, inferior longitudinal fasciculus). |
| gProjection Fibres FA/MD | PCA of 6 bilateral tracts (3 bilateral, 3 unilateral); Forceps major, forceps minor, corticospinal tract, acoustic radiation, medial lemniscus, middle cerebellar peduncle. |
| gThalamic Radiations FA/MD | PCA of 3 bilateral tracts; Superior thalamic radiation, posterior thalamic radiation, anterior thalamic radiation |
| Individual Tracts | All of the above individually. |

***S2.2.2B Variance explained by the first principal component for DTI PCA***

For each global and grouped DTI FA and MD tract measure, the variance explained by the first principal component.

| **PCA** | **FA** | **MD** |
| --- | --- | --- |
| gTotal | 37.1% | 37.6% |
| Association Fibres | 44.6% | 50% |
| Projection Fibres | 35.3% | 29.5% |
| Thalamic Radiations | 61.0% | 71.5% |

***S2.3 Quality Control
S2.3.1 Mental health traits***

***S2.3.1A Conditions excluded for mental health traits***Conditions excluded per participant for final mental health trait phenotypes and data-field the4y correspond to. Response variable corresponds to UKB data coding.

| **Exclusion Type** | **Response variable** |
| --- | --- |
| ***Neuropsychiatric conditions (UKB Data-Field 20002)*** |  |
| Stroke | 1081 |
| Transient ischaemic attack | 1082 |
| Subdural haemorrhage/ Haematoma | 1083 |
| Subarachnoid haemorrhage | 1086 |
| Neurological injury/trauma | 1240 |
| Infection of nervous system | 1244 |
| Brain abscess/Intracranial abscess | 1245 |
| Meningitis | 1247 |
| Chronic/degenerative neurological problem | 1258 |
| Motor neurone disease | 1259 |
| Multiple sclerosis | 1261 |
| Dementia/Alzheimers/Cognitive impairment | 1263 |
| Migraine | 1265 |
| Head injury | 1266 |
| Other demyelinating disease | 1397 |
| Cerebral aneurysm | 1425 |
| Cerebral palsy | 1433 |
| Other neurological problem | 1434 |
| Brain haemorrhage | 1491 |
| Spina bifida | 1524 |
| Ischaemic stroke | 1583 |
| Fracture skull / head | 1626 |
| Meningioma / Benign meningeal tumour | 1659 |
| Benign neuroma | 1683 |
| ***Sleep conditions (UKB Data-Field 20002)*** |  |
| Insomnia | 1616 |
| Sleep apnoea | 1123 |
| ***Cancer (UKB Data-Field 20001)*** |  |
| Meningeal cancer / malignant meningioma | 1032 |
| Brain cancer / primary malignant brain tumour | 1031 |
| ***Shift Work (UKB Data-Field 826)*** |  |
| Job involving shift work | 2, 3 |

***S2.3.1B Sample size per mental health traits post-exclusions***

Sample size for each (*n*=4) mental health trait phenotype after grouping and exclusions.

| **Mental Health Trait** | **Total N** |
| --- | --- |
| Probable Recurrent MDD | 32,285 |
| Probable Single Episode MDD | 13,721 |
| Unipolar Mania | 1,229 |
| Bipolar Depression | 5,278 |
| Control | 85,075 |

***S2.4 Multiple testing correction***

***S2.4.1 Multiple testing corrections applied per neuroimaging measure modality***
Number of neuroimaging measures False Discovery Rate (FDR) was applied for.

| **Measure name** | **Number of measures FDR was applied for** | **Measure name** | **Number of measures FDR was applied for** |
| --- | --- | --- | --- |
| **Bilateral** |  | **Unilateral** |  |
| Individual Cortical Area | 31 | Global Cortical Area |  |
| Individual Mean Cortical Thickness | 31 | Global Mean Cortical Thickness |  |
| Individual Cortical Volume | 31 | Global Cortical Volume |  |
|  | | Lobar Cortical Area | 5 |
|  |  | Lobar Cortical Thickness | 5 |
|  |  | Lobar Cortical Volume | 5 |
| Individual Subcortical Volume | 7 |  | |
| FA Individual | 15 | Global FA |  |
| MD Individual | 15 | Global MD |  |
|  | | FA tract bundles | 3 |
|  |  | MD tract bundles | 3 |

***S2.5 Secondary phenotype demographic tables for brain imaging measure associations***

***S2.5.1 Demographic table for Probable Major Depressive Disorder secondary phenotype***

|  | Control (N=11346) | Case (N=5970) |
| --- | --- | --- |
| **Sex** |  |  |
| Female | 5142 (45.3%) | 3954 (66.2%) |
| Male | 6204 (54.7%) | 2016 (33.8%) |
| **Age (years)** |  |  |
| Mean (SD) | 65.0 (7.39) | 62.4 (7.18) |
| Median [Min, Max] | 66.0 [46.0, 82.0] | 62.0 [45.0, 81.0] |
| **Ethnicity** |  |  |
| Prefer not to answer | 21 (0.2%) | 10 (0.2%) |
| Do not know | 0 (0%) | 2 (0.0%) |
| White | 5 (0.0%) | 2 (0.0%) |
| Mixed | 0 (0%) | 0 (0%) |
| Asian or Asian British | 0 (0%) | 0 (0%) |
| Black or Black British | 0 (0%) | 0 (0%) |
| Chinese | 3 (0.0%) | 1 (0.0%) |
| Other ethnic group | 13 (0.1%) | 13 (0.2%) |
| British | 10971 (96.7%) | 5701 (95.5%) |
| Irish | 170 (1.5%) | 101 (1.7%) |
| Any other white background | 93 (0.8%) | 104 (1.7%) |
| White and Black Caribbean | 10 (0.1%) | 5 (0.1%) |
| White and Black African | 2 (0.0%) | 3 (0.1%) |
| White and Asian | 6 (0.1%) | 4 (0.1%) |
| Any other mixed background | 7 (0.1%) | 6 (0.1%) |
| Indian | 17 (0.1%) | 6 (0.1%) |
| Pakistani | 0 (0%) | 2 (0.0%) |
| Bangladeshi | 0 (0%) | 1 (0.0%) |
| Any other Asian background | 0 (0%) | 1 (0.0%) |
| Caribbean | 22 (0.2%) | 8 (0.1%) |
| African | 6 (0.1%) | 0 (0%) |
| Any other Black background | 0 (0%) | 0 (0%) |
| **Month of Birth** |  |  |
| January | 929 (8.2%) | 498 (8.3%) |
| February | 909 (8.0%) | 485 (8.1%) |
| March | 1025 (9.0%) | 561 (9.4%) |
| April | 985 (8.7%) | 551 (9.2%) |
| May | 956 (8.4%) | 482 (8.1%) |
| June | 959 (8.5%) | 513 (8.6%) |
| July | 957 (8.4%) | 491 (8.2%) |
| August | 951 (8.4%) | 493 (8.3%) |
| September | 941 (8.3%) | 470 (7.9%) |
| October | 942 (8.3%) | 481 (8.1%) |
| November | 882 (7.8%) | 456 (7.6%) |
| December | 910 (8.0%) | 489 (8.2%) |
| **Birth Location Cluster** |  |  |
| 1 | 2094 (18.5%) | 1102 (18.5%) |
| 2 | 4043 (35.6%) | 2147 (36.0%) |
| 3 | 4218 (37.2%) | 2288 (38.3%) |
| 4 | 991 (8.7%) | 433 (7.3%) |

***S2.5.2 Demographic table for summer and winter born Probable Major Depressive Disorder secondary phenotype.***
WP-MDD., Winter born Probable Major Depressive Disorder; SP-MDD., Summer born Probable Major Depressive Disorder.

|  | WP-MDD (N=1472) | SP-MDD (N=1497) |
| --- | --- | --- |
| **Sex** |  |  |
| Female | 980 (66.6%) | 966 (64.5%) |
| Male | 492 (33.4%) | 531 (35.5%) |
| **Age (years)** |  |  |
| Mean (SD) | 62.5 (7.31) | 62.4 (7.11) |
| Median [Min, Max] | 63.0 [45.0, 80.0] | 63.0 [47.0, 81.0] |
| **Ethnicity** |  |  |
| Prefer not to answer | 3 (0.2%) | 1 (0.1%) |
| Do not know | 0 (0%) | 1 (0.1%) |
| White | 1 (0.1%) | 1 (0.1%) |
| Mixed | 0 (0%) | 0 (0%) |
| Asian or Asian British | 0 (0%) | 0 (0%) |
| Black or Black British | 0 (0%) | 0 (0%) |
| Chinese | 0 (0%) | 0 (0%) |
| Other ethnic group | 3 (0.2%) | 2 (0.1%) |
| British | 1410 (95.8%) | 1420 (94.9%) |
| Irish | 20 (1.4%) | 32 (2.1%) |
| Any other white background | 27 (1.8%) | 32 (2.1%) |
| White and Black Caribbean | 1 (0.1%) | 1 (0.1%) |
| White and Black African | 0 (0%) | 2 (0.1%) |
| White and Asian | 2 (0.1%) | 0 (0%) |
| Any other mixed background | 1 (0.1%) | 1 (0.1%) |
| Indian | 1 (0.1%) | 1 (0.1%) |
| Pakistani | 0 (0%) | 1 (0.1%) |
| Bangladeshi | 0 (0%) | 0 (0%) |
| Any other Asian background | 1 (0.1%) | 0 (0%) |
| Caribbean | 2 (0.1%) | 2 (0.1%) |
| African | 0 (0%) | 0 (0%) |
| Any other Black background | 0 (0%) | 0 (0%) |
| **Month of Birth** |  |  |
| January | 498 (33.8%) | 0 (0%) |
| February | 485 (32.9%) | 0 (0%) |
| March | 0 (0%) | 0 (0%) |
| April | 0 (0%) | 0 (0%) |
| May | 0 (0%) | 0 (0%) |
| June | 0 (0%) | 513 (34.3%) |
| July | 0 (0%) | 491 (32.8%) |
| August | 0 (0%) | 493 (32.9%) |
| September | 0 (0%) | 0 (0%) |
| October | 0 (0%) | 0 (0%) |
| November | 0 (0%) | 0 (0%) |
| December | 489 (33.2%) | 0 (0%) |
| **Birth Location Cluster** |  |  |
| 1 | 254 (17.3%) | 261 (17.4%) |
| 2 | 518 (35.2%) | 564 (37.7%) |
| 3 | 595 (40.4%) | 556 (37.1%) |
| 4 | 105 (7.1%) | 116 (7.7%) |

***S2.5.3 Demographic table Probable Recurrent Major Depressive Disorder and Probable Single episode Major Depressive disorder secondary phenotype***

P-SEMDD., Probable Single episode Major Depressive Disorder; P-RMDD., Probable Recurrent Major Depressive Disorder.

|  | P-SEMDD (N=1934) | P-RMDD (N=4036) |
| --- | --- | --- |
| **Sex** |  |  |
| Female | 1274 (65.9%) | 2680 (66.4%) |
| Male | 660 (34.1%) | 1356 (33.6%) |
| **Age (years)** |  |  |
| Mean (SD) | 62.7 (7.11) | 62.2 (7.21) |
| Median [Min, Max] | 63.0 [45.0, 81.0] | 62.0 [45.0, 81.0] |
| **Ethnicity** |  |  |
| Prefer not to answer | 3 (0.2%) | 7 (0.2%) |
| Do not know | 2 (0.1%) | 0 (0%) |
| White | 1 (0.1%) | 1 (0.0%) |
| Mixed | 0 (0%) | 0 (0%) |
| Asian or Asian British | 0 (0%) | 0 (0%) |
| Black or Black British | 0 (0%) | 0 (0%) |
| Chinese | 0 (0%) | 1 (0.0%) |
| Other ethnic group | 2 (0.1%) | 11 (0.3%) |
| British | 1853 (95.8%) | 3848 (95.3%) |
| Irish | 31 (1.6%) | 70 (1.7%) |
| Any other white background | 31 (1.6%) | 73 (1.8%) |
| White and Black Caribbean | 3 (0.2%) | 2 (0.0%) |
| White and Black African | 1 (0.1%) | 2 (0.0%) |
| White and Asian | 3 (0.2%) | 1 (0.0%) |
| Any other mixed background | 0 (0%) | 6 (0.1%) |
| Indian | 2 (0.1%) | 4 (0.1%) |
| Pakistani | 0 (0%) | 2 (0.0%) |
| Bangladeshi | 0 (0%) | 1 (0.0%) |
| Any other Asian background | 0 (0%) | 1 (0.0%) |
| Caribbean | 2 (0.1%) | 6 (0.1%) |
| African | 0 (0%) | 0 (0%) |
| Any other Black background | 0 (0%) | 0 (0%) |
| **Month of Birth** |  |  |
| January | 175 (9.0%) | 323 (8.0%) |
| February | 149 (7.7%) | 336 (8.3%) |
| March | 176 (9.1%) | 385 (9.5%) |
| April | 183 (9.5%) | 368 (9.1%) |
| May | 149 (7.7%) | 333 (8.3%) |
| June | 163 (8.4%) | 350 (8.7%) |
| July | 161 (8.3%) | 330 (8.2%) |
| August | 181 (9.4%) | 312 (7.7%) |
| September | 135 (7.0%) | 335 (8.3%) |
| October | 158 (8.2%) | 323 (8.0%) |
| November | 150 (7.8%) | 306 (7.6%) |
| December | 154 (8.0%) | 335 (8.3%) |
| **Birth Location Cluster** |  |  |
| 1 | 351 (18.1%) | 751 (18.6%) |
| 2 | 693 (35.8%) | 1454 (36.0%) |
| 3 | 768 (39.7%) | 1520 (37.7%) |
| 4 | 122 (6.3%) | 311 (7.7%) |

**SUPPLEMENTARY RESULTS**

***S3. Brain Imaging Measures Supplementary Results***

***S3.1.1 Global T1 measures***

Global T1 brain measure associations with Seasonality.

p-uncorr., p-uncorrected value; p-corr., FDR p-corrected value; S.E., standard error.

| **Brain Imaging Measure** | **Effect Size (β)** | **S.E.** | **t statistic** | **p-uncorr.** | **p-corr** |
| --- | --- | --- | --- | --- | --- |
| GlobalSurfaceArea | -0.002 | 0.004 | -0.41 | 0.682 | - |
| GlobalCorticalVolume | -0.002 | 0.004 | -0.348 | 0.728 | - |
| GlobalCorticalThickness | 0.001 | 0.004 | 0.211 | 0.833 | - |

***S3.1.2 Lobar T1 measures***

Lobar T1 brain measure associations with Seasonality.

p-uncorr., p-uncorrected value; p-corr., FDR p-corrected value; S.E., standard error.

| **Brain Imaging Measure** | **Effect Size (β)** | **S.E.** | **t statistic** | **p-uncorr.** | **p-corr** |
| --- | --- | --- | --- | --- | --- |
| ***Area*** |  |  |  |  |  |
| OccipitalArea | -0.006 | 0.005 | -1.266 | 0.205 | 0.257 |
| ParietalArea | 0.007 | 0.005 | 1.566 | 0.117 | 0.214 |
| CingulateArea | -0.007 | 0.005 | -1.521 | 0.128 | 0.214 |
| TemporalArea | 0 | 0.004 | 0.005 | 0.996 | 0.996 |
| FrontalArea | -0.007 | 0.004 | -1.61 | 0.107 | 0.214 |
| ***Volume*** |  |  |  |  |  |
| OccipitalVolume | -0.002 | 0.005 | -0.406 | 0.685 | 0.685 |
| ParietalVolume | 0.002 | 0.005 | 0.43 | 0.667 | 0.685 |
| CingulateVolume | -0.006 | 0.005 | -1.262 | 0.207 | 0.382 |
| TemporalVolume | 0.005 | 0.004 | 1.203 | 0.229 | 0.382 |
| FrontalVolume | -0.008 | 0.004 | -1.765 | 0.078 | 0.382 |
| ***Thickness*** |  |  |  |  |  |
| OccipitalThickness | 0.013 | 0.005 | 2.436 | 0.015 | **0.037** |
| ParietalThickness | -0.007 | 0.005 | -1.305 | 0.192 | 0.24 |
| CingulateThickness | 0.008 | 0.005 | 1.416 | 0.157 | 0.24 |
| TemporalThickness | 0.014 | 0.005 | 2.628 | 0.009 | **0.037** |
| FrontalThickness | -0.001 | 0.005 | -0.289 | 0.772 | 0.772 |

***S3.1.3 Individual T1 measures***

Individual T1 brain measure associations with Seasonality.

p-uncorr., p-uncorrected value; p-corr., FDR p-corrected value; S.E., standard error; DF., degrees of freedom

| **Brain Imaging Measure** | **Effect Size (β)** | **S.E.** | **DF** | **t statistic** | **p-uncorr.** | **p-corr** |
| --- | --- | --- | --- | --- | --- | --- |
| ***Surface area*** |  |  |  |  |  |  |
| Areaoftransversetemporal | -0.009 | 0.005 | 33196 | -2.07 | 0.038 | 0.268 |
| Areaofsupramarginal | 0.003 | 0.006 | 33196 | 0.533 | 0.594 | 0.764 |
| Areaofsuperiortemporal | -0.006 | 0.006 | 33196 | -1.021 | 0.307 | 0.569 |
| Areaofsuperiorparietal | 0.003 | 0.007 | 33196 | 0.432 | 0.666 | 0.764 |
| Areaofsuperiorfrontal | -0.011 | 0.005 | 33196 | -1.939 | 0.053 | 0.272 |
| Areaofrostralmiddlefrontal | -0.003 | 0.006 | 33196 | -0.545 | 0.586 | 0.764 |
| Areaofrostralanteriorcingulate | -0.002 | 0.004 | 33196 | -0.459 | 0.646 | 0.764 |
| Areaofprecuneus | 0.006 | 0.006 | 33196 | 0.921 | 0.357 | 0.582 |
| Areaofprecentral | -0.006 | 0.006 | 33196 | -1.026 | 0.305 | 0.569 |
| Areaofposteriorcingulate | -0.006 | 0.006 | 33196 | -1.003 | 0.316 | 0.569 |
| Areaofpostcentral | 0.005 | 0.006 | 33196 | 0.795 | 0.427 | 0.661 |
| Areaofpericalcarine | -0.009 | 0.007 | 33196 | -1.3 | 0.194 | 0.505 |
| Areaofparstriangularis | -0.001 | 0.006 | 33196 | -0.099 | 0.921 | 0.963 |
| Areaofparsorbitalis | -0.009 | 0.006 | 33196 | -1.503 | 0.133 | 0.457 |
| Areaofparsopercularis | 0.01 | 0.006 | 33196 | 1.592 | 0.111 | 0.431 |
| Areaofparahippocampal | -0.013 | 0.006 | 33196 | -2.021 | 0.043 | 0.268 |
| Areaofparacentral | -0.008 | 0.006 | 33196 | -1.278 | 0.201 | 0.505 |
| Areaofmiddletemporal | 0.007 | 0.006 | 33196 | 1.108 | 0.268 | 0.569 |
| Areaofmedialorbitofrontal | -0.007 | 0.006 | 33196 | -1.249 | 0.212 | 0.505 |
| Areaoflingual | -0.003 | 0.007 | 33196 | -0.448 | 0.654 | 0.764 |
| Areaoflateralorbitofrontal | -0.001 | 0.006 | 33196 | -0.186 | 0.852 | 0.943 |
| Areaoflateraloccipital | 0 | 0.006 | 33196 | -0.042 | 0.967 | 0.967 |
| Areaofisthmuscingulate | 0.003 | 0.006 | 33196 | 0.508 | 0.612 | 0.764 |
| Areaofinsula | -0.009 | 0.006 | 33196 | -1.437 | 0.151 | 0.467 |
| Areaofinferiortemporal | 0.006 | 0.006 | 33196 | 0.973 | 0.331 | 0.569 |
| Areaofinferiorparietal | 0.012 | 0.006 | 33196 | 2.202 | 0.028 | 0.268 |
| Areaoffusiform | -0.001 | 0.006 | 33196 | -0.086 | 0.932 | 0.963 |
| Areaofentorhinal | 0.011 | 0.006 | 33196 | 1.771 | 0.077 | 0.339 |
| Areaofcuneus | -0.015 | 0.007 | 33196 | -2.292 | 0.022 | 0.268 |
| Areaofcaudalmiddlefrontal | -0.004 | 0.006 | 33196 | -0.575 | 0.565 | 0.764 |
| Areaofcaudalanteriorcingulate | -0.011 | 0.005 | 33196 | -2.444 | 0.015 | 0.268 |
| ***Thickness*** |  |  |  |  |  |  |
| Meanthicknessoftransversetemporal | -0.001 | 0.007 | 33196 | -0.105 | 0.916 | 0.916 |
| Meanthicknessofsupramarginal | -0.004 | 0.007 | 33196 | -0.624 | 0.532 | 0.718 |
| Meanthicknessofsuperiortemporal | 0.014 | 0.006 | 33196 | 2.26 | 0.024 | 0.246 |
| Meanthicknessofsuperiorparietal | -0.011 | 0.007 | 33196 | -1.581 | 0.114 | 0.429 |
| Meanthicknessofsuperiorfrontal | -0.001 | 0.006 | 33196 | -0.173 | 0.862 | 0.891 |
| Meanthicknessofrostralmiddlefrontal | -0.002 | 0.007 | 33196 | -0.307 | 0.759 | 0.871 |
| Meanthicknessofrostralanteriorcingulate | 0.006 | 0.006 | 33196 | 0.915 | 0.36 | 0.589 |
| Meanthicknessofprecuneus | -0.007 | 0.007 | 33196 | -1.053 | 0.292 | 0.589 |
| Meanthicknessofprecentral | -0.006 | 0.007 | 33196 | -0.864 | 0.388 | 0.592 |
| Meanthicknessofposteriorcingulate | -0.006 | 0.006 | 33196 | -0.962 | 0.336 | 0.589 |
| Meanthicknessofpostcentral | -0.01 | 0.007 | 33196 | -1.482 | 0.138 | 0.429 |
| Meanthicknessofpericalcarine | 0.009 | 0.007 | 33196 | 1.41 | 0.159 | 0.447 |
| Meanthicknessofparstriangularis | 0.003 | 0.006 | 33196 | 0.427 | 0.669 | 0.798 |
| Meanthicknessofparsorbitalis | 0.007 | 0.006 | 33196 | 1.135 | 0.256 | 0.589 |
| Meanthicknessofparsopercularis | -0.005 | 0.006 | 33196 | -0.839 | 0.401 | 0.592 |
| Meanthicknessofparahippocampal | 0.014 | 0.007 | 33196 | 2.081 | 0.037 | 0.29 |
| Meanthicknessofparacentral | 0.002 | 0.007 | 33196 | 0.255 | 0.799 | 0.878 |
| Meanthicknessofmiddletemporal | 0.015 | 0.006 | 33196 | 2.353 | 0.019 | 0.246 |
| Meanthicknessofmedialorbitofrontal | 0.003 | 0.006 | 33196 | 0.504 | 0.614 | 0.794 |
| Meanthicknessoflingual | 0.013 | 0.007 | 33196 | 1.974 | 0.048 | 0.298 |
| Meanthicknessoflateralorbitofrontal | -0.007 | 0.007 | 33196 | -1.076 | 0.282 | 0.589 |
| Meanthicknessoflateraloccipital | 0.003 | 0.007 | 33196 | 0.444 | 0.657 | 0.798 |
| Meanthicknessofisthmuscingulate | 0.01 | 0.006 | 33196 | 1.535 | 0.125 | 0.429 |
| Meanthicknessofinsula | 0.007 | 0.007 | 33196 | 1.027 | 0.304 | 0.589 |
| Meanthicknessofinferiortemporal | 0.01 | 0.007 | 33196 | 1.498 | 0.134 | 0.429 |
| Meanthicknessofinferiorparietal | -0.004 | 0.007 | 33196 | -0.634 | 0.526 | 0.718 |
| Meanthicknessoffusiform | 0.016 | 0.007 | 33196 | 2.383 | 0.017 | 0.246 |
| Meanthicknessofentorhinal | 0.001 | 0.006 | 33196 | 0.225 | 0.822 | 0.878 |
| Meanthicknessofcuneus | 0.013 | 0.007 | 33196 | 1.899 | 0.058 | 0.298 |
| Meanthicknessofcaudalmiddlefrontal | -0.006 | 0.007 | 33196 | -0.914 | 0.361 | 0.589 |
| Meanthicknessofcaudalanteriorcingulate | 0.007 | 0.006 | 33196 | 1.15 | 0.25 | 0.589 |
| ***Volume*** |  |  |  |  |  |  |
| Volumeoftransversetemporal | -0.007 | 0.006 | 33196 | -1.341 | 0.18 | 0.728 |
| Volumeofsupramarginal | 0.001 | 0.006 | 33196 | 0.148 | 0.883 | 0.943 |
| Volumeofsuperiortemporal | 0.002 | 0.006 | 33196 | 0.302 | 0.763 | 0.927 |
| Volumeofsuperiorparietal | -0.004 | 0.007 | 33196 | -0.56 | 0.576 | 0.869 |
| Volumeofsuperiorfrontal | -0.012 | 0.006 | 33196 | -2.092 | 0.036 | 0.728 |
| Volumeofrostralmiddlefrontal | -0.003 | 0.006 | 33196 | -0.473 | 0.636 | 0.881 |
| Volumeofrostralanteriorcingulate | -0.002 | 0.004 | 33196 | -0.449 | 0.653 | 0.881 |
| Volumeofprecuneus | -0.001 | 0.006 | 33196 | -0.158 | 0.875 | 0.943 |
| Volumeofprecentral | -0.007 | 0.006 | 33196 | -1.077 | 0.282 | 0.728 |
| Volumeofposteriorcingulate | -0.011 | 0.006 | 33196 | -1.804 | 0.071 | 0.728 |
| Volumeofpostcentral | 0 | 0.006 | 33196 | -0.036 | 0.971 | 0.971 |
| Volumeofpericalcarine | -0.005 | 0.007 | 33196 | -0.794 | 0.427 | 0.828 |
| Volumeofparstriangularis | 0.001 | 0.006 | 33196 | 0.109 | 0.913 | 0.943 |
| Volumeofparsorbitalis | -0.002 | 0.006 | 33196 | -0.283 | 0.777 | 0.927 |
| Volumeofparsopercularis | 0.007 | 0.006 | 33196 | 1.053 | 0.292 | 0.728 |
| Volumeofparahippocampal | -0.004 | 0.007 | 33196 | -0.592 | 0.554 | 0.869 |
| Volumeofparacentral | -0.006 | 0.006 | 33196 | -0.93 | 0.352 | 0.728 |
| Volumeofmiddletemporal | 0.008 | 0.006 | 33196 | 1.355 | 0.175 | 0.728 |
| Volumeofmedialorbitofrontal | -0.006 | 0.006 | 33196 | -0.938 | 0.348 | 0.728 |
| Volumeoflingual | 0.002 | 0.007 | 33196 | 0.282 | 0.778 | 0.927 |
| Volumeoflateralorbitofrontal | -0.003 | 0.006 | 33196 | -0.55 | 0.582 | 0.869 |
| Volumeoflateraloccipital | -0.001 | 0.006 | 33196 | -0.154 | 0.877 | 0.943 |
| Volumeofisthmuscingulate | 0.009 | 0.006 | 33196 | 1.47 | 0.142 | 0.728 |
| Volumeofinsula | -0.003 | 0.006 | 33196 | -0.541 | 0.589 | 0.869 |
| Volumeofinferiortemporal | 0.007 | 0.006 | 33196 | 1.094 | 0.274 | 0.728 |
| Volumeofinferiorparietal | 0.01 | 0.005 | 33196 | 1.816 | 0.069 | 0.728 |
| Volumeoffusiform | 0.004 | 0.006 | 33196 | 0.634 | 0.526 | 0.869 |
| Volumeofentorhinal | 0.008 | 0.006 | 33196 | 1.293 | 0.196 | 0.728 |
| Volumeofcuneus | -0.006 | 0.006 | 33196 | -0.944 | 0.345 | 0.728 |
| Volumeofcaudalmiddlefrontal | -0.006 | 0.006 | 33196 | -0.939 | 0.348 | 0.728 |
| Volumeofcaudalanteriorcingulate | -0.007 | 0.005 | 33196 | -1.517 | 0.129 | 0.728 |

***S3.1.4 Subcortical Measures***

Subcortical brain measure associations with Seasonality.

p-uncorr., p-uncorrected value; p-corr., FDR p-corrected value; S.E., standard error; DF., degrees of freedom

| **Brain Imaging Measure** | **Effect Size (β)** | **S.E.** | **DF** | **t statistic** | **p-uncorr.** | **p-corr** |
| --- | --- | --- | --- | --- | --- | --- |
| ***Volume*** |  |  |  |  |  |  |
| VolumeOfthalamus | -0.003 | 0.006 | 33196 | -0.535 | 0.593 | 0.935 |
| VolumeOfputamen | 0 | 0.006 | 33196 | 0.069 | 0.945 | 0.945 |
| VolumeOfpallidum | 0.003 | 0.007 | 33196 | 0.443 | 0.657 | 0.935 |
| VolumeOfhippocampus | -0.003 | 0.006 | 33196 | -0.459 | 0.646 | 0.935 |
| VolumeOfcaudate | 0.003 | 0.007 | 33196 | 0.43 | 0.668 | 0.935 |
| VolumeOfamygdala | 0.012 | 0.006 | 33196 | 2.023 | 0.043 | 0.301 |
| VolumeOfaccumbens | -0.001 | 0.006 | 33196 | -0.221 | 0.825 | 0.945 |

***S3.1.5 DTI Global Measures***

Global DTI brain measure associations with Seasonality.

p-uncorr., p-uncorrected value; p-corr., FDR p-corrected value; S.E., standard error.

| **Brain Imaging Measure** | **Effect Size (β)** | **S.E.** | **t statistic** | **p-uncorr.** | **p-corr** |
| --- | --- | --- | --- | --- | --- |
| MDTotalTracts | -0.001 | 0.005 | -0.279 | 0.78 | **-** |
| **FATotalTracts** | **-0.017** | **0.005** | **-3.177** | **0.001** | - |

***S3.1.6 DTI Grouped Tract Measures***

Grouped DTI tract brain measure associations with Seasonality.

p-uncorr., p-uncorrected value; p-corr., FDR p-corrected value; S.E., standard error.

| **Brain Imaging Measure** | **Effect Size (β)** | **S.E.** | **t statistic** | **p-uncorr.** | **p-corr** |
| --- | --- | --- | --- | --- | --- |
| ***FA*** |  |  |  |  |  |
| **FAThalamicRadiations** | **-0.014** | **0.005** | **-2.717** | **0.007** | **0.01** |
| FAProjectionFibres | -0.003 | 0.005 | -0.51 | 0.61 | 0.61 |
| **FAAssociationFibres** | **-0.022** | **0.005** | **-4.153** | **3.285-05** | **9.855^-05^** |
| ***MD*** |  |  |  |  |  |
| MDThalamicRadiations | -0.003 | 0.005 | -0.671 | 0.502 | 0.776 |
| MDProjectionFibres | 0.001 | 0.005 | 0.165 | 0.869 | 0.869 |
| MDAssociationFibres | 0.003 | 0.005 | 0.647 | 0.517 | 0.776 |

***S3.1.7 DTI Individual Tract Measures***

Individual DTI tract brain measure associations with Seasonality.

p-uncorr., p-uncorrected value; p-corr., FDR p-corrected value; S.E., standard error; DF., degrees of freedom

| **Brain Imaging Measure** | **Effect Size (β)** | **S.E.** | **DF** | **t statistic** | **p-uncorr.** | **p-corr** |
| --- | --- | --- | --- | --- | --- | --- |
| ***FA*** |  |  |  |  |  |  |
| **FAintractanteriorthalamicradiation** | **-0.016** | **0.007** | **33197** | **-2.348** | **0.019** | **0.047** |
| **FAintractposteriorthalamicradiation** | **-0.019** | **0.007** | **33197** | **-2.708** | **0.007** | **0.034** |
| FAintractsuperiorthalamicradiation | 0.005 | 0.007 | 33197 | 0.722 | 0.47 | 0.641 |
| FAintractmediallemniscus | 0.011 | 0.006 | 33197 | 1.644 | 0.1 | 0.17 |
| FAintractacousticradiation | -0.004 | 0.006 | 33197 | -0.599 | 0.549 | 0.686 |
| FAintractcorticospinaltract | 0.001 | 0.007 | 33197 | 0.205 | 0.838 | 0.884 |
| **FAintractinferiorlongitudinalfasciculus** | **-0.018** | **0.007** | **33197** | **-2.586** | **0.01** | **0.036** |
| FAintractuncinatefasciculus | -0.001 | 0.007 | 33197 | -0.18 | 0.857 | 0.884 |
| FAintractsuperiorlongitudinalfasciculus | -0.001 | 0.007 | 33197 | -0.146 | 0.884 | 0.884 |
| **FAintractinferiorfrontooccipitalfasciculus** | **-0.021** | **0.007** | **33197** | **-3.029** | **0.002** | **0.018** |
| FAintractparahippocampalpartofcingulum | -0.012 | 0.007 | 33197 | -1.859 | 0.063 | 0.135 |
| **FAintractcingulategyruspartofcingulum** | **-0.02** | **0.006** | **33197** | **-3.44** | **0.001** | **0.009** |
| FAintractforcepsminor | -0.006 | 0.005 | NA | -1.198 | 0.231 | 0.346 |
| **FAintractforcepsmajor** | **-0.013** | **0.005** | **NA** | **-2.373** | **0.018** | **0.047** |
| **FAintractmiddlecerebellarpeduncle** | **-0.009** | **0.005** | **NA** | **-1.635** | **0.102** | **0.17** |
| ***MD*** |  |  |  |  |  |  |
| MDintractanteriorthalamicradiation | -0.004 | 0.006 | 33197 | -0.646 | 0.519 | 0.636 |
| MDintractposteriorthalamicradiation | -0.006 | 0.006 | 33197 | -0.981 | 0.326 | 0.544 |
| MDintractsuperiorthalamicradiation | 0.009 | 0.006 | 33197 | 1.464 | 0.143 | 0.537 |
| MDintractmediallemniscus | 0.009 | 0.006 | 33197 | 1.495 | 0.135 | 0.537 |
| MDintractacousticradiation | -0.009 | 0.006 | 33197 | -1.333 | 0.183 | 0.544 |
| MDintractcorticospinaltract | -0.001 | 0.007 | 33197 |  | 0.858 | 0.858 |
| MDintractinferiorlongitudinalfasciculus | -0.004 | 0.007 | 33197 | -0.669 | 0.504 | 0.636 |
| MDintractuncinatefasciculus | -0.002 | 0.007 | 33197 | -0.31 | 0.756 | 0.81 |
| MDintractsuperiorlongitudinalfasciculus | -0.008 | 0.007 | 33197 | -1.12 | 0.263 | 0.544 |
| MDintractinferiorfrontooccipitalfasciculus | -0.008 | 0.007 | 33197 | -1.125 | 0.261 | 0.544 |
| MDintractparahippocampalpartofcingulum | 0.01 | 0.006 | 33197 | 1.546 | 0.122 | 0.537 |
| MDintractcingulategyruspartofcingulum | 0.005 | 0.007 | 33197 | 0.718 | 0.473 | 0.636 |
| MDintractforcepsminor | -0.01 | 0.005 | NA | -1.907 | 0.057 | 0.537 |
| MDintractforcepsmajor | -0.005 | 0.005 | NA | -0.997 | 0.319 | 0.544 |
| MDintractmiddlecerebellarpeduncle | 0.003 | 0.005 | NA | 0.596 | 0.551 | 0.636 |

***S3.2 Seasonality associations with brain imaging measures covarying for birth weight***

***S3.2.1 Global T1 measures***

Global T1 brain measure associations with Seasonality.

p-uncorr., p-uncorrected value; p-corr., FDR p-corrected value; S.E., standard error.

| **Brain Imaging Measure** | **Effect Size (β)** | **S.E.** | **t statistic** | **p-uncorr.** | **p-corr** |
| --- | --- | --- | --- | --- | --- |
| GlobalCorticalThickness | 0.001 | 0.004 | 0.211 | 0.833 | - |
| GlobalCorticalVolume | -0.002 | 0.004 | -0.348 | 0.728 | - |
| GlobalSurfaceArea | -0.002 | 0.004 | -0.41 | 0.682 | - |

***S3.2.2 Lobar T1 measures***

Lobar T1 brain measure associations with Seasonality.

p-uncorr., p-uncorrected value; p-corr., FDR p-corrected value; S.E., standard error.

| **Brain Imaging Measure** | **Effect Size (β)** | **S.E.** | **t statistic** | **p-uncorr.** | **p-corr** |
| --- | --- | --- | --- | --- | --- |
| ***Area*** |  |  |  |  |  |
| OccipitalArea | -0.011 | 0.006 | -1.8 | 0.072 | 0.29 |
| ParietalArea | 0.004 | 0.006 | 0.772 | 0.44 | 0.55 |
| CingulateArea | -0.008 | 0.006 | -1.372 | 0.17 | 0.29 |
| TemporalArea | 0.001 | 0.005 | 0.274 | 0.784 | 0.784 |
| FrontalArea | -0.007 | 0.005 | -1.36 | 0.174 | 0.29 |
| ***Volume*** |  |  |  |  |  |
| OccipitalVolume | 0.001 | 0.006 | 0.242 | 0.809 | 0.809 |
| ParietalVolume | 0.005 | 0.006 | 0.848 | 0.396 | 0.744 |
| CingulateVolume | -0.003 | 0.006 | -0.5 | 0.617 | 0.771 |
| TemporalVolume | 0.012 | 0.005 | 2.21 | 0.027 | 0.136 |
| FrontalVolume | -0.004 | 0.005 | -0.761 | 0.447 | 0.744 |
| ***Thickness*** |  |  |  |  |  |
| **OccipitalThickness** | **0.025** | **0.007** | **3.747** | **0.0002** | **0.0004** |
| ParietalThickness | 0.003 | 0.006 | 0.436 | 0.663 | 0.663 |
| **CingulateThickness** | **0.015** | **0.007** | **2.282** | **0.022** | **0.037** |
| **TemporalThickness** | **0.025** | **0.007** | **3.856** | **0.0001** | **0.0004** |
| FrontalThickness | 0.006 | 0.006 | 0.953 | 0.34 | 0.425 |

***S3.2.3 Individual T1 measures***

Individual T1 brain measure associations with Seasonality.

p-uncorr., p-uncorrected value; p-corr., FDR p-corrected value; S.E., standard error; DF., degrees of freedom

| **Brain Imaging Measure** | **Effect Size (β)** | **S.E.** | **DF** | **t statistic** | **p-uncorr.** | **p-corr** |
| --- | --- | --- | --- | --- | --- | --- |
| ***Surface area*** |  |  |  |  |  |  |
| Areaoftransversetemporal | -0.008 | 0.006 | 21400 | -1.413 | 0.158 | 0.563 |
| Areaofsupramarginal | 0.008 | 0.007 | 21400 | 1.015 | 0.31 | 0.601 |
| Areaofsuperiortemporal | -0.005 | 0.007 | 21400 | -0.673 | 0.501 | 0.647 |
| Areaofsuperiorparietal | 0.002 | 0.008 | 21400 | 0.213 | 0.832 | 0.921 |
| Areaofsuperiorfrontal | -0.01 | 0.007 | 21400 | -1.513 | 0.13 | 0.563 |
| Areaofrostralmiddlefrontal | -0.001 | 0.008 | 21400 | -0.074 | 0.941 | 0.941 |
| Areaofrostralanteriorcingulate | -0.001 | 0.005 | 21400 | -0.13 | 0.897 | 0.927 |
| Areaofprecuneus | 0.006 | 0.008 | 21400 | 0.822 | 0.411 | 0.647 |
| Areaofprecentral | -0.009 | 0.008 | 21400 | -1.232 | 0.218 | 0.563 |
| Areaofposteriorcingulate | -0.008 | 0.008 | 21400 | -1.073 | 0.283 | 0.601 |
| Areaofpostcentral | 0.002 | 0.008 | 21400 | 0.292 | 0.77 | 0.884 |
| Areaofpericalcarine | -0.015 | 0.009 | 21400 | -1.755 | 0.079 | 0.563 |
| Areaofparstriangularis | -0.001 | 0.008 | 21400 | -0.13 | 0.897 | 0.927 |
| Areaofparsorbitalis | -0.008 | 0.008 | 21400 | -1.039 | 0.299 | 0.601 |
| Areaofparsopercularis | 0.012 | 0.008 | 21400 | 1.557 | 0.119 | 0.563 |
| Areaofparahippocampal | -0.011 | 0.008 | 21400 | -1.362 | 0.173 | 0.563 |
| Areaofparacentral | -0.015 | 0.008 | 21400 | -1.867 | 0.062 | 0.563 |
| Areaofmiddletemporal | 0.01 | 0.008 | 21400 | 1.242 | 0.214 | 0.563 |
| Areaofmedialorbitofrontal | -0.01 | 0.007 | 21400 | -1.379 | 0.168 | 0.563 |
| Areaoflingual | -0.006 | 0.008 | 21400 | -0.729 | 0.466 | 0.647 |
| Areaoflateralorbitofrontal | 0.002 | 0.008 | 21400 | 0.306 | 0.759 | 0.884 |
| Areaoflateraloccipital | -0.006 | 0.008 | 21400 | -0.759 | 0.448 | 0.647 |
| Areaofisthmuscingulate | 0.005 | 0.007 | 21400 | 0.698 | 0.485 | 0.647 |
| Areaofinsula | -0.007 | 0.008 | 21400 | -0.871 | 0.384 | 0.647 |
| Areaofinferiortemporal | 0.008 | 0.007 | 21400 | 1.126 | 0.26 | 0.601 |
| Areaofinferiorparietal | 0.005 | 0.007 | 21400 | 0.679 | 0.497 | 0.647 |
| Areaoffusiform | -0.004 | 0.007 | 21400 | -0.531 | 0.595 | 0.738 |
| Areaofentorhinal | 0.01 | 0.008 | 21400 | 1.243 | 0.214 | 0.563 |
| Areaofcuneus | -0.013 | 0.008 | 21400 | -1.662 | 0.097 | 0.563 |
| Areaofcaudalmiddlefrontal | -0.007 | 0.008 | 21400 | -0.881 | 0.378 | 0.647 |
| Areaofcaudalanteriorcingulate | -0.014 | 0.006 | 21400 | -2.532 | 0.011 | 0.352 |
| ***Thickness*** |  |  |  |  |  |  |
| Meanthicknessoftransversetemporal | 0.009 | 0.008 | 21400 | 1.063 | 0.288 | 0.558 |
| Meanthicknessofsupramarginal | 0.002 | 0.008 | 21400 | 0.24 | 0.811 | 0.93 |
| Meanthicknessofsuperiortemporal | 0.022 | 0.008 | 21400 | 2.772 | 0.006 | 0.056 |
| Meanthicknessofsuperiorparietal | -0.001 | 0.008 | 21400 | -0.164 | 0.87 | 0.93 |
| Meanthicknessofsuperiorfrontal | 0.003 | 0.008 | 21400 | 0.418 | 0.676 | 0.873 |
| Meanthicknessofrostralmiddlefrontal | 0.004 | 0.008 | 21400 | 0.438 | 0.661 | 0.873 |
| Meanthicknessofrostralanteriorcingulate | 0.013 | 0.008 | 21400 | 1.665 | 0.096 | 0.27 |
| Meanthicknessofprecuneus | -0.002 | 0.008 | 21400 | -0.204 | 0.838 | 0.93 |
| Meanthicknessofprecentral | 0.001 | 0.008 | 21400 | 0.079 | 0.937 | 0.968 |
| Meanthicknessofposteriorcingulate | -0.005 | 0.008 | 21400 | -0.694 | 0.487 | 0.793 |
| Meanthicknessofpostcentral | -0.003 | 0.008 | 21400 | -0.315 | 0.753 | 0.93 |
| Meanthicknessofpericalcarine | 0.017 | 0.008 | 21400 | 2.03 | 0.042 | 0.164 |
| Meanthicknessofparstriangularis | 0.004 | 0.008 | 21400 | 0.51 | 0.61 | 0.873 |
| Meanthicknessofparsorbitalis | 0.006 | 0.008 | 21400 | 0.698 | 0.485 | 0.793 |
| Meanthicknessofparsopercularis | -0.001 | 0.008 | 21400 | -0.168 | 0.867 | 0.93 |
| Meanthicknessofparahippocampal | 0.018 | 0.008 | 21400 | 2.263 | 0.024 | 0.122 |
| Meanthicknessofparacentral | 0.009 | 0.008 | 21400 | 1.081 | 0.28 | 0.558 |
| Meanthicknessofmiddletemporal | 0.024 | 0.008 | 21400 | 3.102 | 0.002 | 0.055 |
| Meanthicknessofmedialorbitofrontal | 0.011 | 0.008 | 21400 | 1.418 | 0.156 | 0.404 |
| Meanthicknessoflingual | 0.023 | 0.008 | 21400 | 2.688 | 0.007 | 0.056 |
| Meanthicknessoflateralorbitofrontal | 0 | 0.008 | 21400 | 0.009 | 0.992 | 0.992 |
| Meanthicknessoflateraloccipital | 0.016 | 0.009 | 21400 | 1.868 | 0.062 | 0.193 |
| Meanthicknessofisthmuscingulate | 0.009 | 0.008 | 21400 | 1.111 | 0.267 | 0.558 |
| Meanthicknessofinsula | 0.008 | 0.008 | 21400 | 0.992 | 0.321 | 0.586 |
| Meanthicknessofinferiortemporal | 0.015 | 0.008 | 21400 | 1.864 | 0.062 | 0.193 |
| Meanthicknessofinferiorparietal | 0.005 | 0.008 | 21400 | 0.656 | 0.512 | 0.793 |
| Meanthicknessoffusiform | 0.024 | 0.008 | 21400 | 2.917 | 0.004 | 0.055 |
| Meanthicknessofentorhinal | 0.009 | 0.008 | 21400 | 1.163 | 0.245 | 0.558 |
| Meanthicknessofcuneus | 0.019 | 0.008 | 21400 | 2.287 | 0.022 | 0.122 |
| Meanthicknessofcaudalmiddlefrontal | 0.004 | 0.008 | 21400 | 0.465 | 0.642 | 0.873 |
| Meanthicknessofcaudalanteriorcingulate | 0.015 | 0.007 | 21400 | 2.086 | 0.037 | 0.164 |
| ***Volume*** |  |  |  |  |  |  |
| Volumeoftransversetemporal | -0.001 | 0.007 | 21400 | -0.198 | 0.843 | 0.98 |
| Volumeofsupramarginal | 0.008 | 0.007 | 21400 | 1.116 | 0.265 | 0.928 |
| Volumeofsuperiortemporal | 0.007 | 0.008 | 21400 | 0.89 | 0.374 | 0.928 |
| Volumeofsuperiorparietal | 0 | 0.008 | 21400 | 0.026 | 0.98 | 0.98 |
| Volumeofsuperiorfrontal | -0.008 | 0.007 | 21400 | -1.221 | 0.222 | 0.928 |
| Volumeofrostralmiddlefrontal | 0.002 | 0.008 | 21400 | 0.239 | 0.811 | 0.98 |
| Volumeofrostralanteriorcingulate | 0.002 | 0.005 | 21400 | 0.344 | 0.731 | 0.98 |
| Volumeofprecuneus | 0.004 | 0.008 | 21400 | 0.466 | 0.641 | 0.98 |
| Volumeofprecentral | -0.005 | 0.008 | 21400 | -0.63 | 0.529 | 0.928 |
| Volumeofposteriorcingulate | -0.012 | 0.008 | 21400 | -1.54 | 0.123 | 0.903 |
| Volumeofpostcentral | 0.002 | 0.008 | 21400 | 0.25 | 0.803 | 0.98 |
| Volumeofpericalcarine | -0.006 | 0.009 | 21400 | -0.698 | 0.485 | 0.928 |
| Volumeofparstriangularis | 0.001 | 0.007 | 21400 | 0.138 | 0.89 | 0.98 |
| Volumeofparsorbitalis | -0.001 | 0.008 | 21400 | -0.067 | 0.947 | 0.98 |
| Volumeofparsopercularis | 0.01 | 0.008 | 21400 | 1.261 | 0.207 | 0.928 |
| Volumeofparahippocampal | 0.003 | 0.008 | 21400 | 0.351 | 0.726 | 0.98 |
| Volumeofparacentral | -0.007 | 0.008 | 21400 | -0.949 | 0.343 | 0.928 |
| Volumeofmiddletemporal | 0.016 | 0.008 | 21400 | 2.052 | 0.04 | 0.903 |
| Volumeofmedialorbitofrontal | -0.005 | 0.007 | 21400 | -0.615 | 0.539 | 0.928 |
| Volumeoflingual | 0.006 | 0.008 | 21400 | 0.679 | 0.497 | 0.928 |
| Volumeoflateralorbitofrontal | 0.003 | 0.008 | 21400 | 0.417 | 0.677 | 0.98 |
| Volumeoflateraloccipital | 0.001 | 0.008 | 21400 | 0.096 | 0.923 | 0.98 |
| Volumeofisthmuscingulate | 0.011 | 0.008 | 21400 | 1.455 | 0.146 | 0.903 |
| Volumeofinsula | 0 | 0.008 | 21400 | -0.055 | 0.956 | 0.98 |
| Volumeofinferiortemporal | 0.013 | 0.008 | 21400 | 1.644 | 0.1 | 0.903 |
| Volumeofinferiorparietal | 0.006 | 0.007 | 21400 | 0.906 | 0.365 | 0.928 |
| Volumeoffusiform | 0.005 | 0.007 | 21400 | 0.671 | 0.502 | 0.928 |
| Volumeofentorhinal | 0.012 | 0.008 | 21400 | 1.556 | 0.12 | 0.903 |
| Volumeofcuneus | 0 | 0.008 | 21400 | -0.059 | 0.953 | 0.98 |
| Volumeofcaudalmiddlefrontal | -0.006 | 0.008 | 21400 | -0.709 | 0.478 | 0.928 |
| Volumeofcaudalanteriorcingulate | -0.006 | 0.006 | 21400 | -1.01 | 0.312 | 0.928 |

***S3.2.4 Subcortical Measures***

Subcortical T1 brain measure associations with Seasonality.

p-uncorr., p-uncorrected value; p-corr., FDR p-corrected value; S.E., standard error; DF., degrees of freedom

| **Brain Imaging Measure** | **Effect Size (β)** | **S.E.** | **DF** | **t statistic** | **p-uncorr.** | **p-corr** |
| --- | --- | --- | --- | --- | --- | --- |
| ***Volume*** |  |  |  |  |  |  |
| VolumeOfthalamus | -0.002 | 0.008 | 21400 | -0.293 | 0.769 | 0.769 |
| VolumeOfputamen | 0.006 | 0.008 | 21400 | 0.819 | 0.413 | 0.697 |
| VolumeOfpallidum | 0.005 | 0.008 | 21400 | 0.595 | 0.552 | 0.697 |
| VolumeOfhippocampus | 0.004 | 0.008 | 21400 | 0.563 | 0.573 | 0.697 |
| VolumeOfcaudate | 0.009 | 0.008 | 21400 | 1.01 | 0.312 | 0.697 |
| **VolumeOfamygdala** | **0.021** | **0.008** | **21400** | **2.747** | **0.006** | **0.042** |
| VolumeOfaccumbens | -0.004 | 0.007 | 21400 | -0.528 | 0.598 | 0.697 |

***S3.2.5 DTI Global Measures***

Global DTI tract brain measure associations with Seasonality.

p-uncorr., p-uncorrected value; p-corr., FDR p-corrected value; S.E., standard error.

| **Brain Imaging Measure** | **Effect Size (β)** | **S.E.** | **t statistic** | **p-uncorr.** | **p-corr** |
| --- | --- | --- | --- | --- | --- |
| MDTotalTracts | -0.001 | 0.005 | -0.279 | 0.78 | - |
| **FATotalTracts** | **-0.017** | **0.005** | **-3.177** | **0.001** | - |

***S3.2.6 DTI Grouped Tract Measures***

Grouped DTI tract brain measure associations with Seasonality.

p-uncorr., p-uncorrected value; p-corr., FDR p-corrected value; S.E., standard error.

| **Brain Imaging Measure** | **Effect Size (β)** | **S.E.** | **t statistic** | **p-uncorr.** | **p-corr** |
| --- | --- | --- | --- | --- | --- |
| ***FA*** |  |  |  |  |  |
| **FAThalamicRadiations** | **-0.022** | **0.006** | **-3.357** | **0.001** | **0.001** |
| FAProjectionFibres | -0.006 | 0.007 | -0.908 | 0.364 | 0.364 |
| **FAAssociationFibres** | **-0.028** | **0.006** | **-4.383** | **1.173-05** | **3.520-05** |
| ***MD*** |  |  |  |  |  |
| MDThalamicRadiations | 0.002 | 0.006 | 0.317 | 0.751 | 0.751 |
| MDProjectionFibres | 0.011 | 0.006 | 1.696 | 0.09 | 0.27 |
| MDAssociationFibres | 0.008 | 0.006 | 1.232 | 0.218 | 0.327 |

***S3.2.7 DTI Individual Tract Measures***

Individual DTI tract brain measure associations with Seasonality.

p-uncorr., p-uncorrected value; p-corr., FDR p-corrected value; S.E., standard error; DF., degrees of freedom

| **Brain Imaging Measure** | **Effect Size (β)** | **S.E.** | **DF** | **t statistic** | **p-uncorr.** | **p-corr** |
| --- | --- | --- | --- | --- | --- | --- |
| ***FA*** |  |  |  |  |  |  |
| **FAintractanteriorthalamicradiation** | **-0.026** | **0.009** | **21401** | **-3.021** | **0.003** | **0.008** |
| **FAintractposteriorthalamicradiation** | **-0.027** | **0.008** | **21401** | **-3.128** | **0.002** | **0.007** |
| FAintractsuperiorthalamicradiation | 0.007 | 0.009 | 21401 | 0.752 | 0.452 | 0.522 |
| FAintractmediallemniscus | 0.012 | 0.008 | 21401 | 1.519 | 0.129 | 0.241 |
| FAintractacousticradiation | -0.009 | 0.008 | 21401 | -1.188 | 0.235 | 0.316 |
| FAintractcorticospinaltract | 0.003 | 0.009 | 21401 | 0.302 | 0.763 | 0.763 |
| **FAintractinferiorlongitudinalfasciculus** | **-0.022** | **0.008** | **21401** | **-2.635** | **0.008** | **0.021** |
| FAintractuncinatefasciculus | -0.01 | 0.008 | 21401 | -1.241 | 0.215 | 0.316 |
| FAintractsuperiorlongitudinalfasciculus | -0.004 | 0.008 | 21401 | -0.517 | 0.605 | 0.649 |
| **FAintractinferiorfrontooccipitalfasciculus** | **-0.027** | **0.008** | **21401** | **-3.174** | **0.002** | **0.007** |
| FAintractparahippocampalpartofcingulum | -0.01 | 0.008 | 21401 | -1.227 | 0.22 | 0.316 |
| **FAintractcingulategyruspartofcingulum** | **-0.025** | **0.007** | **21401** | **-3.458** | **0.001** | **0.007** |
| **FAintractforcepsminor** | **-0.015** | **0.006** | **NA** | **-2.315** | **0.021** | **0.044** |
| **FAintractforcepsmajor** | **-0.021** | **0.007** | **NA** | **-3.141** | **0.002** | **0.007** |
| FAintractmiddlecerebellarpeduncle | -0.008 | 0.007 | NA | -1.143 | 0.253 | 0.316 |
| ***MD*** |  |  |  |  |  |  |
| MDintractanteriorthalamicradiation | 0.003 | 0.008 | 21401 | 0.339 | 0.735 | 0.787 |
| MDintractposteriorthalamicradiation | -0.004 | 0.008 | 21401 | -0.453 | 0.651 | 0.751 |
| MDintractsuperiorthalamicradiation | 0.012 | 0.008 | 21401 | 1.585 | 0.113 | 0.403 |
| MDintractmediallemniscus | 0.017 | 0.008 | 21401 | 2.27 | 0.023 | 0.348 |
| MDintractacousticradiation | -0.004 | 0.008 | 21401 | -0.539 | 0.59 | 0.751 |
| MDintractcorticospinaltract | 0.004 | 0.008 | 21401 | 0.48 | 0.631 | 0.751 |
| MDintractinferiorlongitudinalfasciculus | -0.005 | 0.008 | 21401 | -0.564 | 0.573 | 0.751 |
| MDintractuncinatefasciculus | 0.002 | 0.008 | 21401 | 0.25 | 0.803 | 0.803 |
| MDintractsuperiorlongitudinalfasciculus | -0.007 | 0.008 | 21401 | -0.778 | 0.437 | 0.751 |
| MDintractinferiorfrontooccipitalfasciculus | -0.006 | 0.008 | 21401 | -0.698 | 0.485 | 0.751 |
| MDintractparahippocampalpartofcingulum | 0.014 | 0.008 | 21401 | 1.759 | 0.079 | 0.393 |
| MDintractcingulategyruspartofcingulum | 0.012 | 0.008 | 21401 | 1.498 | 0.134 | 0.403 |
| MDintractforcepsminor | -0.012 | 0.006 | NA | -1.937 | 0.053 | 0.393 |
| MDintractforcepsmajor | 0.003 | 0.007 | NA | 0.512 | 0.609 | 0.751 |
| MDintractmiddlecerebellarpeduncle | 0.008 | 0.007 | NA | 1.259 | 0.208 | 0.52 |

***S3.3. Seasonality associations with mental health traits***

Mental health traits associated with seasonality under four sets of logistic regression models testing a total of four covariate sets per mental health trait. p-uncorr = p-uncorrected value; p-corr = Bonferroni p-corrected value; S.E = standard error.

| **Mental Health Trait** | **Covariates** | **Effect Size(β) / Log(OR)** | **S.E.** | **p-uncorr** | **p-corr** |
| --- | --- | --- | --- | --- | --- |
| **Recurrent Depression** | **Phenotype+sex+Age+centre** | **0.025** | **0.01** | **0.01** | **0.038** |
| **Recurrent Depression** | **Phenotype+sex+Age+centre+BirthLocation** | **0.024** | **0.01** | **0.01** | **0.042** |
| **Recurrent Depression** | **Phenotype+sex+Age+centre+BirthLocation+Townsend** | **0.026** | **0.01** | **0.007** | **0.028** |
| **Recurrent Depression** | **Phenotype+sex+Age+centre+BirthLocation+Townsend+Age2** | **0.026** | **0.01** | **0.007** | **0.028** |
| Single Depression | Phenotype+sex+Age+centre | 0.016 | 0.013 | 0.239 | 0.956 |
| Single Depression | Phenotype+sex+Age+centre+BirthLocation | 0.016 | 0.013 | 0.243 | 0.971 |
| Single Depression | Phenotype+sex+Age+centre+BirthLocation+Townsend | 0.016 | 0.013 | 0.218 | 0.874 |
| Single Depression | Phenotype+sex+Age+centre+BirthLocation+Townsend+Age2 | 0.017 | 0.013 | 0.212 | 0.847 |
| Unipolar Mania | Phenotype+sex+Age+centre | 0.064 | 0.041 | 0.12 | 0.479 |
| Unipolar Mania | Phenotype+sex+Age+centre+BirthLocation | 0.065 | 0.041 | 0.115 | 0.458 |
| Unipolar Mania | Phenotype+sex+Age+centre+BirthLocation+Townsend | 0.065 | 0.041 | 0.11 | 0.44 |
| Unipolar Mania | Phenotype+sex+Age+centre+BirthLocation+Townsend+Age2 | 0.066 | 0.041 | 0.108 | 0.432 |
| Bipolar Depression | Phenotype+sex+Age+centre | 0.006 | 0.02 | 0.762 | 1 |
| Bipolar Depression | Phenotype+sex+Age+centre+BirthLocation | 0.006 | 0.02 | 0.76 | 1 |
| Bipolar Depression | Phenotype+sex+Age+centre+BirthLocation+Townsend | 0.009 | 0.02 | 0.673 | 1 |
| Bipolar Depression | Phenotype+sex+Age+centre+BirthLocation+Townsend+Age2 | 0.009 | 0.02 | 0.665 | 1 |

***S3.4 Mental health trait associations with brain imaging measures***

***S3.4.1 Global T1 measures***
Effect sizes for mental health traits associations with global T1 brain imaging measures. P-RMDD., Probable Recurrent Major Depressive Disorder; P-SEMDD., Probable Single episode Major Depressive Disorder; P-BD., Probable Bipolar Depression; P-UM., Probable Unipolar Mania. * represents FDR corrected p-value <0.05.

| **Model** | **P-RMDD** | **P-SEMDD** | **P-BD** | **P-UM** |
| --- | --- | --- | --- | --- |
| Global Surface Area | -0.006 | -0.044* | -0.046 | 0.04 |
| Global Cortical Volume | -0.009 | -0.048* | -0.042 | -0.026 |
| Global Cortical Thickness | -0.006 | -0.044* | -0.044 | 0.04 |

***S3.4.2 Lobar T1 measures***

Effect sizes for mental health traits associations with lobar T1 brain imaging measures. P-RMDD., Probable Recurrent Major Depressive Disorder; P-SEMDD., Probable Single episode Major Depressive Disorder; P-BD., Probable Bipolar Depression; P-UM., Probable Unipolar Mania. * represents FDR corrected p-value <0.05.

| **Model** | **P-RMDD** | **P-SEMDD** | **P-BD** | **P-UM** |
| --- | --- | --- | --- | --- |
| Frontal Area | -0.005 | -0.042 | -0.05 | 0.04 |
| Frontal Thickness | -0.011 | -0.024 | -0.044 | -0.105 |
| Frontal Volume | -0.011 | -0.053* | -0.064 | -0.013 |
| Temporal Area | -0.005 | -0.034 | -0.041 | 0.024 |
| Temporal Thickness | -0.006 | 0.004 | 0.04 | -0.102 |
| Temporal Volume | -0.017 | -0.039 | -0.037 | -0.053 |
| Cingulate Area | 0.016 | -0.021 | 0.02 | 0.046 |
| Cingulate Thickness | -0.017 | -0.006 | 0.023 | 0.045 |
| Cingulate Volume | 0 | -0.037 | 0.03 | 0.049 |
| Parietal Area | -0.009 | -0.049 | -0.054 | 0.008 |
| Parietal Thickness | 0.007 | 0.004 | 0.028 | -0.117 |
| Parietal Volume | -0.007 | -0.045 | -0.038 | -0.058 |
| Occipital Area | -0.006 | -0.039 | -0.033 | 0.098 |
| Occipital Thickness | 0.035 | 0.002 | 0.03 | -0.062 |
| Occipital Volume | 0.013 | -0.03 | -0.009 | 0.048 |

***S3.4.3 Individual T1 measures***

Effect sizes for mental health traits associations with individual T1 brain imaging measures. P-RMDD., Probable Recurrent Major Depressive Disorder; P-SEMDD., Probable Single episode Major Depressive Disorder; P-BD., Probable Bipolar Depression; P-UM., Probable Unipolar Mania. * represents FDR corrected p-value <0.05.

| **Model** | **P-RMDD** | **P-SEMDD** | **P-BD** | **P-UM** |
| --- | --- | --- | --- | --- |
| Area of caudal anterior cingulate | 0.004 | -0.003 | 0.012 | -0.005 |
| Area of caudal middle frontal | -0.004 | -0.019 | -0.023 | 0.013 |
| Area of cuneus | -0.015 | -0.054 | -0.044 | -0.013 |
| Area of entorhinal | -0.007 | -0.042 | 0.012 | 0.001 |
| Area of fusiform | -0.014 | -0.031 | -0.042 | -0.001 |
| Area of inferior parietal | -0.011 | -0.016 | -0.059 | -0.026 |
| Area of inferior temporal | -0.005 | -0.037 | -0.047 | 0.007 |
| Area of insula | -0.012 | -0.031 | -0.012 | 0.076 |
| Area of isthmus cingulate | 0.026 | -0.008 | 0.027 | 0.059 |
| Area of lateraloccipital | 0.01 | -0.013 | -0.015 | 0.122 |
| Area of lateralorbitofrontal | -0.001 | -0.05 | -0.045 | 0.07 |
| Area of lingual | -0.006 | -0.029 | -0.027 | 0.085 |
| Area of medial orbitofrontal | -0.017 | -0.044 | -0.021 | 0.059 |
| Area of middle temporal | -0.005 | -0.014 | -0.053 | 0.001 |
| Area of paracentral | -0.003 | -0.039 | -0.063 | 0.081 |
| Area of parahippocampal | 0.008 | -0.013 | -0.037 | 0.07 |
| Area of parsopercularis | 0.006 | -0.007 | 0.016 | -0.006 |
| Area of parsorbitalis | -0.009 | -0.035 | -0.016 | 0.098 |
| Area of parstriangularis | -0.007 | -0.028 | 0.009 | 0 |
| Area of pericalcarine | -0.03 | -0.056 | -0.036 | 0.065 |
| Area of postcentral | -0.006 | -0.044 | -0.054 | 0.051 |
| Area of posterior cingulate | 0.001 | -0.014 | -0.007 | 0.007 |
| Area of precentral | -0.008 | -0.032 | -0.073 | 0.04 |
| Area of precuneus | -0.003 | -0.049 | -0.01 | 0.012 |
| Area of rostralanterior cingulate | 0.011 | -0.025 | 0.016 | 0.055 |
| Area of rostralmiddle frontal | -0.001 | -0.036 | -0.039 | 0.001 |
| Area of superior frontal | -0.003 | -0.029 | -0.042 | 0.028 |
| Area of superior parietal | -0.01 | -0.049 | -0.029 | 0.048 |
| Area of superior temporal | 0.007 | -0.022 | -0.011 | 0.027 |
| Area of supramarginal | -0.003 | -0.027 | -0.03 | -0.071 |
| Area of transversetemporal | -0.004 | -0.02 | 0.006 | 0.036 |
| Volume of caudal anterior cingulate | -0.009 | -0.014 | 0.025 | 0.015 |
| Volume of caudal middle frontal | 0 | -0.031 | -0.032 | -0.009 |
| Volume of cuneus | -0.003 | -0.046 | -0.022 | 0.001 |
| Volume of entorhinal | 0.007 | -0.039 | 0.02 | 0.038 |
| Volume of fusiform | -0.028 | -0.046 | -0.051 | -0.04 |
| Volume of inferior parietal | -0.01 | -0.019 | -0.055 | -0.066 |
| Volume of inferior temporal | -0.015 | -0.042 | -0.057 | -0.039 |
| Volume of insula | -0.036 | -0.044 | -0.036 | 0.008 |
| Volume of isthmus cingulate | 0.03 | -0.003 | 0.05 | 0.062 |
| Volume of lateraloccipital | 0.022 | 0.001 | 0.015 | 0.034 |
| Volume of lateralorbitofrontal | -0.009 | -0.053 | -0.064 | 0.071 |
| Volume of lingual | 0.011 | -0.039 | -0.019 | 0.063 |
| Volume of medial orbitofrontal | -0.041 | -0.062 | -0.084 | 0.005 |
| Volume of middle temporal | -0.013 | -0.012 | -0.045 | -0.062 |
| Volume of paracentral | -0.008 | -0.041 | -0.069 | 0.049 |
| Volume of parahippocampal | 0.011 | -0.005 | 0.041 | 0.002 |
| Volume of parsopercularis | 0.003 | -0.018 | 0 | -0.018 |
| Volume of parsorbitalis | 0.001 | -0.032 | -0.009 | 0.018 |
| Volume of parstriangularis | -0.006 | -0.032 | 0.005 | -0.018 |
| Volume of pericalcarine | -0.016 | -0.05 | -0.041 | 0.047 |
| Volume of postcentral | 0.005 | -0.019 | -0.028 | -0.016 |
| Volume of posterior cingulate | -0.012 | -0.028 | 0.002 | 0.01 |
| Volume of precentral | -0.015 | -0.034 | -0.079 | -0.023 |
| Volume of precuneus | -0.002 | -0.049 | 0.005 | -0.017 |
| Volume of rostralanterior cingulate | 0 | -0.035 | 0.005 | 0.036 |
| Volume of rostralmiddle frontal | -0.001 | -0.043 | -0.035 | -0.043 |
| Volume of superior frontal | -0.01 | -0.04 | -0.052 | -0.023 |
| Volume of superior parietal | -0.001 | -0.046 | 0.016 | -0.006 |
| Volume of superior temporal | -0.003 | -0.022 | 0.006 | -0.054 |
| Volume of supramarginal | -0.013 | -0.029 | -0.039 | -0.12 |
| Volume of transversetemporal | 0.013 | 0 | 0.036 | -0.038 |

***S3.4.4 Subcortical Measures***

Effect sizes for mental health traits associations with subcortical brain imaging measures. P-RMDD., Probable Recurrent Major Depressive Disorder; P-SEMDD., Probable Single episode Major Depressive Disorder; P-BD., Probable Bipolar Depression; P-UM., Probable Unipolar Mania. * represents FDR corrected p-value <0.05.

| **Model** | **P-RMDD** | **P-SEMDD** | **P-BD** | **P-UM** |
| --- | --- | --- | --- | --- |
| Volume Of accumbens | -0.033 | -0.014 | -0.01 | -0.003 |
| Volume Of amygdala | 0.008 | 0.014 | 0.066 | -0.031 |
| Volume Of caudate | 0.023 | -0.022 | 0.048 | 0.059 |
| Volume Of hippocampus | 0.004 | 0.005 | 0.074 | -0.001 |
| Volume Of pallidum | -0.022 | -0.027 | -0.023 | 0.043 |
| Volume Of putamen | -0.006 | -0.026 | 0.061 | 0.007 |
| Volume Of thalamus | -0.044* | -0.045 | -0.025 | -0.037 |

***S3.4.5 DTI Global Measures***

Effect sizes for mental health traits associations with global DTI brain imaging measures. P-RMDD., Probable Recurrent Major Depressive Disorder; P-SEMDD., Probable Single episode Major Depressive Disorder; P-BD., Probable Bipolar Depression; P-UM., Probable Unipolar Mania. * represents FDR corrected p-value <0.05.

| **Model** | **P-RMDD** | **P-SEMDD** | **P-BD** | **P-UM** |
| --- | --- | --- | --- | --- |
| FA Total Tracts | -0.058* | -0.049* | -0.065 | -0.045 |
| MD Total Tracts | 0.059* | 0.045* | 0.033 | 0.035 |

***S3.4.6 DTI Grouped Tract Measures***

Effect sizes for mental health traits associations with DTI grouped tract brain imaging measures.

P-RMDD., Probable Recurrent Major Depressive Disorder; P-SEMDD., Probable Single episode Major Depressive Disorder; P-BD., Probable Bipolar Depression; P-UM., Probable Unipolar Mania. * represents FDR corrected p-value <0.05.

| **Model** | **P-RMDD** | **P-SEMDD** | **P-BD** | **P-UM** |
| --- | --- | --- | --- | --- |
| FA Association Fibers | -0.058* | -0.046 | -0.039 | -0.024 |
| FA Projection Fibers | -0.012 | -0.032 | -0.056 | -0.059 |
| FA Thalamic Radiations | -0.09* | -0.056 | -0.104* | -0.083 |
| MD Association Fibers | 0.039* | 0.036 | -0.003 | 0.034 |
| MD Projection Fibers | 0.068* | 0.046 | 0.04 | 0.001 |
| MD Thalamic Radiations | 0.072* | 0.044 | 0.073 | 0.079 |
|  |  |  |  |  |

***S3.4.7 DTI Individual Tract Measures***

Effect sizes for mental health traits associations with individual DTI brain imaging measures.

P-RMDD., Probable Recurrent Major Depressive Disorder; P-SEMDD., Probable Single episode Major Depressive Disorder; P-BD., Probable Bipolar Depression; P-UM., Probable Unipolar Mania. * represents FDR corrected p-value <0.05.

| **Model** | **P-RMDD** | **P-SEMDD** | **P-BD** | **P-UM** |
| --- | --- | --- | --- | --- |
| FA cingulate gyrus part of cingulum | -0.033* | -0.023 | 0.001 | -0.004 |
| FA parahippocampal part of cingulum | -0.018 | -0.02 | -0.033 | -0.029 |
| FA inferior frontooccipital fasciculus | -0.048* | -0.048 | -0.05 | -0.058 |
| FA superior longitudinal fasciculus | -0.04* | -0.022 | -0.068 | -0.023 |
| FA uncinate fasciculus | -0.038* | -0.035 | 0.011 | 0.037 |
| FA inferior longitudinal fasciculus | -0.053* | -0.038 | -0.043 | -0.026 |
| FA corticospinal tract | 0.007 | 0.008 | 0.023 | 0.009 |
| FA acoustic radiation | -0.01 | -0.028 | -0.071 | -0.005 |
| FA medial lemniscus | -0.011 | -0.013 | -0.059 | -0.12 |
| FA superior thalamic radiation | -0.052* | -0.032 | -0.039 | 0.011 |
| FA posterior thalamic radiation | -0.092* | -0.054 | -0.12* | -0.11 |
| FA anterior thalamic radiation | -0.058* | -0.04 | -0.067 | -0.075 |
| MD cingulate gyrus part of cingulum | 0.045* | 0.047 | -0.012 | 0.029 |
| MD parahippocampal part of cingulum | 0.009 | 0.019 | -0.012 | 0.046 |
| MD inferior frontooccipital fasciculus | 0.047* | 0.033 | 0.029 | 0.002 |
| MD superior longitudinal fasciculus | 0.049* | 0.027 | 0.046 | 0.024 |
| MD uncinate fasciculus | 0.037* | 0.037 | -0.041 | -0.033 |
| MD inferior longitudinal fasciculus | 0.038* | 0.016 | 0.011 | 0.018 |
| MD corticospinal tract | 0.036* | 0.043 | 0.027 | 0.074 |
| MD acoustic radiation | 0.009 | 0.014 | 0.015 | -0.062 |
| MD medial lemniscus | -0.004 | 0.025 | 0.008 | 0.13 |
| MD superior thalamic radiation | 0.071* | 0.047 | 0.038 | 0.005 |
| MD posterior thalamic radiation | 0.056* | 0.029 | 0.079 | 0.108 |
| MD anterior thalamic radiation | 0.061* | 0.045 | 0.051 | 0.045 |
| FA middle cerebellar peduncle | 0.009 | -0.039 | 0.002 | -0.103 |
| FA forceps major | -0.019 | -0.036 | -0.076 | 0.042 |
| FA forceps minor | -0.047* | -0.038 | -0.073 | -0.04 |
| MD middle cerebellar peduncle | 0.078* | 0.031 | 0.034 | -0.014 |
| MD forceps major | 0.01 | 0.006 | -0.002 | -0.102 |
| MD forceps minor | 0.041* | 0.064 | 0.068 | 0 |

***S.3.5 Secondary phenotype associations with brain imaging measures***

***S3.5.1 Global T1 measures***
Effect sizes for secondary phenotype associations with global T1 brain imaging measures. P-MDD., Probable Major Depressive Disorder; P-SEMDD., SP-MDD vs WP-MDD., summer-born P-RMDD cases compared to winter-born P-RMDD cases; P-RMDD vs P-SEMDD., Probable Recurrent Major Depressive Disorder cases compared to Probable Single episode Major Depressive Disorder cases. * represents FDR corrected p-value <0.05.

| **Model** | **P-MDD** | **SP-MDD vs WP-MDD** | **P-RMDD vs P-SEMDD** |
| --- | --- | --- | --- |
| Global Surface Area | -0.018 | -0.039 | -0.04 |
| Global Cortical Volume | -0.021 | -0.021 | -0.042 |
| Global Cortical Thickness | -0.018 | -0.046 | -0.04 |

***S3.5.2 Lobar T1 measures***
Effect sizes for secondary phenotype associations with lobar T1 brain imaging measures. P-MDD., Probable Major Depressive Disorder; P-SEMDD., SP-MDD vs WP-MDD., summer-born P-RMDD cases compared to winter-born P-RMDD cases; P-RMDD vs P-SEMDD., Probable Recurrent Major Depressive Disorder cases compared to Probable Single episode Major Depressive Disorder cases. * represents FDR corrected p-value <0.05.

| **Model** | **P-MDD** | **SP-MDD vs WP-MDD** | **P-RMDD vs P-SEMDD** |
| --- | --- | --- | --- |
| Frontal Area | -0.017 | -0.022 | -0.038 |
| Frontal Thickness | -0.014 | 0.014 | -0.016 |
| Frontal Volume | -0.024 | -0.01 | -0.045 |
| Temporal Area | -0.014 | -0.048 | -0.03 |
| Temporal Thickness | -0.003 | 0.051 | 0.011 |
| Temporal Volume | -0.024 | -0.023 | -0.023 |
| Cingulate Area | 0.004 | -0.032 | -0.038 |
| Cingulate Thickness | -0.012 | 0.014 | 0.006 |
| Cingulate Volume | -0.011 | -0.027 | -0.039 |
| Parietal Area | -0.022 | -0.038 | -0.041 |
| Parietal Thickness | 0.005 | 0.015 | -0.004 |
| Parietal Volume | -0.019 | -0.028 | -0.04 |
| Occipital Area | -0.016 | -0.043 | -0.036 |
| Occipital Thickness | 0.023 | 0.049 | -0.034 |
| Occipital Volume | -0.001 | -0.018 | -0.048 |

***S3.5.3 Individual T1 measures***

Effect sizes for secondary phenotype associations with individual T1 brain imaging measures.

P-MDD., Probable Major Depressive Disorder; P-SEMDD., SP-MDD vs WP-MDD., summer-born P-RMDD cases compared to winter-born P-RMDD cases; P-RMDD vs P-SEMDD., Probable Recurrent Major Depressive Disorder cases compared to Probable Single episode Major Depressive Disorder cases. * represents FDR corrected p-value <0.05.

| **Model** | **P-MDD** | **SP-MDD vs WP-MDD** | **P-RMDD vs P-SEMDD** |  |
| --- | --- | --- | --- | --- |
| Area of caudal anterior cingulate | 0.003 | -0.014 | -0.009 |  |
| Area of caudal middle frontal | -0.01 | 0.017 | -0.017 |  |
| Area of cuneus | -0.027 | -0.054 | -0.039 |  |
| Area of entorhinal | -0.019 | -0.004 | -0.038 |  |
| Area of fusiform | -0.019 | -0.052 | -0.019 |  |
| Area of inferior parietal | -0.012 | -0.022 | -0.007 |  |
| Area of inferior temporal | -0.015 | -0.037 | -0.034 |  |
| Area of insula | -0.02 | -0.03 | -0.019 |  |
| Area of isthmus cingulate | 0.013 | -0.034 | -0.03 |  |
| Area of lateraloccipital | 0.004 | -0.037 | -0.029 |  |
| Area of lateralorbitofrontal | -0.017 | -0.033 | -0.047 |  |
| Area of lingual | -0.015 | -0.027 | -0.026 |  |
| Area of medial orbitofrontal | -0.026 | -0.039 | -0.028 |  |
| Area of middle temporal | -0.007 | -0.033 | -0.011 |  |
| Area of paracentral | -0.014 | -0.02 | -0.036 |  |
| Area of parahippocampal | 0.001 | -0.045 | -0.021 |  |
| Area of parsopercularis | 0.002 | -0.013 | -0.016 |  |
| Area of parsorbitalis | -0.018 | -0.042 | -0.025 |  |
| Area of parstriangularis | -0.014 | -0.002 | -0.022 |  |
| Area of pericalcarine | -0.039 | -0.022 | -0.024 |  |
| Area of postcentral | -0.018 | -0.028 | -0.04 |  |
| Area of posterior cingulate | -0.003 | -0.027 | -0.016 |  |
| Area of precentral | -0.016 | -0.002 | -0.025 |  |
| Area of precuneus | -0.019 | -0.011 | -0.048 |  |
| Area of rostralanterior cingulate | -0.001 | -0.01 | -0.037 |  |
| Area of rostralmiddle frontal | -0.012 | -0.012 | -0.033 |  |
| Area of superior frontal | -0.012 | -0.029 | -0.028 |  |
| Area of superior parietal | -0.023 | -0.023 | -0.042 |  |
| Area of superior temporal | -0.003 | -0.037 | -0.029 |  |
| Area of supramarginal | -0.011 | -0.06 | -0.021 |  |
| Area of transversetemporal | -0.009 | -0.033 | -0.02 |  |
| Volume of caudal anterior cingulate | -0.009 | -0.015 | -0.009 |  |
| Volume of caudal middle frontal | -0.011 | 0.013 | -0.033 |  |
| Volume of cuneus | -0.016 | -0.037 | -0.042 |  |
| Volume of entorhinal | -0.007 | -0.004 | -0.049 |  |
| Volume of fusiform | -0.033 | -0.035 | -0.018 |  |
| Volume of inferior parietal | -0.013 | -0.009 | -0.011 |  |
| Volume of inferior temporal | -0.023 | -0.021 | -0.029 |  |
| Volume of insula | -0.04 | -0.009 | -0.01 |  |
| Volume of isthmus cingulate | 0.018 | -0.019 | -0.031 |  |
| Volume of lateraloccipital | 0.017 | -0.022 | -0.029 |  |
| Volume of lateralorbitofrontal | -0.023 | -0.027 | -0.044 |  |
| Volume of lingual | -0.007 | 0.003 | -0.05 |  |
| Volume of medial orbitofrontal | -0.046* | -0.024 | -0.023 |  |
| Volume of middle temporal | -0.012 | -0.009 | 0 |  |
| Volume of paracentral | -0.018 | -0.013 | -0.037 |  |
| Volume of parahippocampal | 0.005 | -0.028 | -0.019 |  |
| Volume of parsopercularis | -0.003 | -0.002 | -0.025 |  |
| Volume of parsorbitalis | -0.01 | -0.032 | -0.033 |  |
| Volume of parstriangularis | -0.015 | 0.005 | -0.028 |  |
| Volume of pericalcarine | -0.027 | 0.011 | -0.032 |  |
| Volume of postcentral | -0.003 | -0.018 | -0.027 |  |
| Volume of posterior cingulate | -0.016 | -0.033 | -0.018 |  |
| Volume of precentral | -0.021 | 0.012 | -0.024 |  |
| Volume of precuneus | -0.018 | -0.013 | -0.047 |  |
| Volume of rostralanterior cingulate | -0.011 | -0.003 | -0.035 |  |
| Volume of rostralmiddle frontal | -0.013 | -0.001 | -0.04 |  |
| Volume of superior frontal | -0.019 | -0.019 | -0.033 |  |
| Volume of superior parietal | -0.016 | -0.023 | -0.049 |  |
| Volume of superior temporal | -0.009 | -0.012 | -0.019 |  |
| Volume of supramarginal | -0.018 | -0.042 | -0.014 |  |
| Volume of transversetemporal | 0.008 | -0.025 | -0.015 |  |

***S3.5.4 Subcortical Measures***

Effect sizes for secondary phenotype associations with subcortical brain imaging measures.

P-MDD., Probable Major Depressive Disorder; P-SEMDD., SP-MDD vs WP-MDD., summer-born P-RMDD cases compared to winter-born P-RMDD cases; P-RMDD vs P-SEMDD., Probable Recurrent Major Depressive Disorder cases compared to Probable Single episode Major Depressive Disorder cases. * represents FDR corrected p-value <0.05.

| **Model** | **P-MDD** | **SP-MDD vs WP-MDD** | **P-RMDD vs P-SEMDD** |
| --- | --- | --- | --- |
| Volume Of accumbens | -0.025 | -0.024 | 0.02 |
| Volume Of amygdala | 0.01 | 0.03 | 0.005 |
| Volume Of caudate | 0.007 | -0.01 | -0.037 |
| Volume Of hippocampus | 0.005 | -0.023 | 0 |
| Volume Of pallidum | -0.022 | -0.006 | -0.001 |
| Volume Of putamen | -0.013 | -0.001 | -0.018 |
| Volume Of thalamus | -0.044* | -0.029 | 0.004 |

***S3.5.5 DTI Global Measures***

Effect sizes for secondary phenotype associations with global DTI brain imaging measures.

P-MDD., Probable Major Depressive Disorder; P-SEMDD., SP-MDD vs WP-MDD., summer-born P-RMDD cases compared to winter-born P-RMDD cases; P-RMDD vs P-SEMDD., Probable Recurrent Major Depressive Disorder cases compared to Probable Single episode Major Depressive Disorder cases. * represents FDR corrected p-value <0.05.

| **Model** | **P-MDD** | **SP-MDD vs WP-MDD** | **P-RMDD vs P-SEMDD** |
| --- | --- | --- | --- |
| FA Total Tracts | -0.057* | -0.015 | 0.015 |
| MD Total Tracts | 0.056* | -0.029 | -0.017 |

***S3.5.6 DTI Grouped Tract Measures***

Effect sizes for secondary phenotype associations with DTI grouped tract brain imaging measures.

P-MDD., Probable Major Depressive Disorder; P-SEMDD., SP-MDD vs WP-MDD., summer-born P-RMDD cases compared to winter-born P-RMDD cases; P-RMDD vs P-SEMDD., Probable Recurrent Major Depressive Disorder cases compared to Probable Single episode Major Depressive Disorder cases. * represents FDR corrected p-value <0.05.

| **Model** | **P-MDD** | **SP-MDD vs WP-MDD** | **P-RMDD vs P-SEMDD** |
| --- | --- | --- | --- |
| Frontal Area | -0.017 | -0.022 | -0.038 |
| Frontal Thickness | -0.014 | 0.014 | -0.016 |
| Frontal Volume | -0.024 | -0.01 | -0.045 |
| Temporal Area | -0.014 | -0.048 | -0.03 |
| Temporal Thickness | -0.003 | 0.051 | 0.011 |
| Temporal Volume | -0.024 | -0.023 | -0.023 |
| Cingulate Area | 0.004 | -0.032 | -0.038 |
| Cingulate Thickness | -0.012 | 0.014 | 0.006 |
| Cingulate Volume | -0.011 | -0.027 | -0.039 |
| Parietal Area | -0.022 | -0.038 | -0.041 |
| Parietal Thickness | 0.005 | 0.015 | -0.004 |
| Parietal Volume | -0.019 | -0.028 | -0.04 |
| Occipital Area | -0.016 | -0.043 | -0.036 |
| Occipital Thickness | 0.023 | 0.049 | -0.034 |
| Occipital Volume | -0.001 | -0.018 | -0.048 |

***S3.5.7 DTI Individual Tract Measures***

Effect sizes for secondary phenotype associations with individual DTI brain imaging measures.

P-MDD., Probable Major Depressive Disorder; P-SEMDD., SP-MDD vs WP-MDD., summer-born P-RMDD cases compared to winter-born P-RMDD cases; P-RMDD vs P-SEMDD., Probable Recurrent Major Depressive Disorder cases compared to Probable Single episode Major Depressive Disorder cases. * represents FDR corrected p-value <0.05.

| **Model** | **P-MDD** | **SP-MDD vs WP-MDD** | **P-RMDD vs P-SEMDD** |  |
| --- | --- | --- | --- | --- |
| FA middle cerebellar peduncle | -0.007 | 0.024 | -0.042 |  |
| FA forceps major | -0.025 | 0.027 | -0.014 |  |
| FA forceps minor | -0.045* | 0.003 | 0.011 |  |
| MD middle cerebellar peduncle | 0.063* | -0.009 | -0.048 |  |
| MD forceps major | 0.011 | -0.025 | -0.007 |  |
| MD forceps minor | 0.05* | -0.035 | 0.017 |  |
| FA cingulate gyrus part of cingulum | -0.03* | -0.002 | 0.011 |  |
| FA parahippocampal part of cingulum | -0.02 | 0.019 | 0.004 |  |
| FA inferior frontooccipital fasciculus | -0.049* | -0.03 | 0.003 |  |
| FA superior longitudinal fasciculus | -0.036* | -0.013 | 0.02 |  |
| FA uncinate fasciculus | -0.036* | -0.023 | 0.007 |  |
| FA inferior longitudinal fasciculus | -0.05* | -0.009 | 0.022 |  |
| FA corticospinal tract | 0.004 | -0.044 | 0.006 |  |
| FA acoustic radiation | -0.018 | 0.015 | -0.015 |  |
| FA medial lemniscus | -0.012 | 0.019 | -0.006 |  |
| FA superior thalamic radiation | -0.049* | -0.034 | 0.026 |  |
| FA posterior thalamic radiation | -0.081* | -0.019 | 0.041 |  |
| FA anterior thalamic radiation | -0.054* | -0.036 | 0.022 |  |
| MD cingulate gyrus part of cingulum | 0.048* | -0.023 | -0.003 |  |
| MD parahippocampal part of cingulum | 0.014 | -0.056 | 0.005 |  |
| MD inferior frontooccipital fasciculus | 0.044* | -0.017 | -0.018 |  |
| MD superior longitudinal fasciculus | 0.044* | -0.006 | -0.023 |  |
| MD uncinate fasciculus | 0.037* | -0.01 | -0.001 |  |
| MD inferior longitudinal fasciculus | 0.032* | -0.019 | -0.026 |  |
| MD corticospinal tract | 0.039* | -0.02 | 0.005 |  |
| MD acoustic radiation | 0.011 | -0.024 | 0.004 |  |
| MD medial lemniscus | 0.007 | 0.027 | 0.03 |  |
| MD superior thalamic radiation | 0.064* | 0.007 | -0.026 |  |
| MD posterior thalamic radiation | 0.048* | -0.004 | -0.026 |  |
| MD anterior thalamic radiation | 0.057* | -0.005 | -0.02 |  |

***S3.6. Mental health trait and seasonality interactions with brain imaging measures***

***S3.6.1 Global T1 measures***

Effect sizes for the association of the interaction of mental health traits and seasonality with global T1 brain imaging measures.

P-RMDD., Probable Recurrent Major Depressive Disorder; P-SEMDD., Probable Single episode Major Depressive Disorder; P-BD., Probable Bipolar Depression; P-UM., Probable Unipolar Mania. * represents FDR corrected p-value <0.05.

| **Model** | **P-RMDD** | **P-SEMDD** | **P-BD** | **P-UM** |
| --- | --- | --- | --- | --- |
| Global Surface Area | -0.033 | 0.013 | -0.027 | 0.074 |
| Global Cortical Volume | -0.023 | 0.021 | -0.07 | 0.015 |
| Global Cortical Thickness | -0.038 | 0.001 | -0.028 | 0.06 |

***S3.6.2 Lobar T1 measures***

Effect sizes for the association of the interaction of mental health traits and seasonality with lobar T1 brain imaging measures. P-RMDD., Probable Recurrent Major Depressive Disorder; P-SEMDD., Probable Single episode Major Depressive Disorder; P-BD., Probable Bipolar Depression; P-UM., Probable Unipolar Mania. * represents FDR corrected p-value <0.05.

| **Model** | **P-RMDD** | **P-SEMDD** | **P-BD** | **P-UM** |  |
| --- | --- | --- | --- | --- | --- |
| Frontal Area | -0.019 | 0.032 | -0.019 | 0.093 |  |
| Frontal Thickness | 0.004 | 0.002 | -0.08 | -0.106 |  |
| Frontal Volume | -0.013 | 0.032 | -0.059 | 0.045 |  |
| Temporal Area | -0.031 | 0 | -0.024 | 0.068 |  |
| Temporal Thickness | 0.002 | 0.002 | -0.058 | -0.194 |  |
| Temporal Volume | -0.026 | 0.007 | -0.063 | -0.013 |  |
| Cingulate Area | -0.022 | 0.039 | 0.038 | 0.006 |  |
| Cingulate Thickness | 0.006 | 0.006 | -0.002 | -0.151 |  |
| Cingulate Volume | -0.016 | 0.04 | 0.038 | -0.059 |  |
| Parietal Area | -0.032 | -0.012 | -0.031 | 0.058 |  |
| Parietal Thickness | 0.007 | 0.022 | -0.097 | -0.048 |  |
| Parietal Volume | -0.023 | 0.006 | -0.087 | 0.023 |  |
| Occipital Area | -0.046 | 0.012 | -0.042 | 0.046 |  |
| Occipital Thickness | 0.026 | 0.014 | -0.058 | -0.085 |  |
| Occipital Volume | -0.024 | 0.018 | -0.068 | -0.009 |  |

***S3.6.3 Individual T1 measures***

Effect sizes for the association of the interaction of mental health traits and seasonality with individual T1 brain imaging measures. P-RMDD., Probable Recurrent Major Depressive Disorder; P-SEMDD., Probable Single episode Major Depressive Disorder; P-BD., Probable Bipolar Depression; P-UM., Probable Unipolar Mania. * represents FDR corrected p-value <0.05.

| **Model** | **P-RMDD** | **P-SEMDD** | **P-BD** | **P-UM** |
| --- | --- | --- | --- | --- |
| Area of caudal anterior cingulate | -0.002 | 0.048 | 0.028 | -0.024 |
| Area of caudal middle frontal | -0.015 | 0.03 | -0.008 | 0.119 |
| Area of cuneus | -0.022 | 0.026 | -0.018 | 0.043 |
| Area of entorhinal | -0.028 | -0.016 | -0.042 | 0.052 |
| Area of fusiform | -0.059 | -0.016 | -0.056 | 0.025 |
| Area of inferior parietal | -0.035 | -0.001 | -0.067 | 0.088 |
| Area of inferior temporal | -0.02 | 0.02 | -0.021 | 0.047 |
| Area of insula | 0.001 | -0.012 | -0.01 | 0.022 |
| Area of isthmus cingulate | -0.056 | 0.013 | -0.007 | 0.001 |
| Area of lateraloccipital | -0.039 | 0.008 | -0.041 | 0.092 |
| Area of lateralorbitofrontal | -0.034 | 0.038 | -0.054 | 0.08 |
| Area of lingual | -0.044 | -0.002 | -0.042 | -0.011 |
| Area of medial orbitofrontal | -0.017 | 0.031 | -0.018 | -0.016 |
| Area of middle temporal | -0.02 | 0.017 | 0.016 | 0.085 |
| Area of paracentral | 0.006 | 0.001 | 0.02 | 0.055 |
| Area of parahippocampal | -0.027 | 0 | -0.026 | -0.065 |
| Area of parsopercularis | -0.033 | -0.003 | -0.011 | 0.049 |
| Area of parsorbitalis | -0.014 | 0.006 | -0.006 | 0.019 |
| Area of parstriangularis | -0.004 | -0.009 | 0.003 | 0.044 |
| Area of pericalcarine | -0.039 | 0.015 | -0.024 | -0.02 |
| Area of postcentral | -0.012 | 0.01 | 0.019 | 0.125 |
| Area of posterior cingulate | -0.013 | 0.004 | 0.032 | 0.071 |
| Area of precentral | -0.021 | 0.025 | 0.016 | 0.15 |
| Area of precuneus | -0.021 | -0.015 | -0.007 | -0.016 |
| Area of rostralanterior cingulate | 0.004 | 0.024 | 0.032 | -0.018 |
| Area of rostralmiddle frontal | -0.002 | 0.057 | -0.027 | 0.058 |
| Area of superior frontal | -0.012 | 0.017 | -0.021 | 0.05 |
| Area of superior parietal | -0.028 | -0.004 | -0.027 | -0.079 |
| Area of superior temporal | -0.019 | -0.013 | -0.023 | 0.062 |
| Area of supramarginal | -0.025 | -0.041 | -0.029 | 0.075 |
| Area of transversetemporal | -0.017 | -0.026 | -0.006 | 0.123 |
| Volume of caudal anterior cingulate | -0.005 | 0.039 | 0.02 | -0.067 |
| Volume of caudal middle frontal | -0.018 | 0.03 | -0.049 | 0.073 |
| Volume of cuneus | -0.019 | 0.02 | -0.048 | -0.016 |
| Volume of entorhinal | -0.038 | -0.001 | -0.037 | -0.071 |
| Volume of fusiform | -0.056 | -0.023 | -0.098 | 0.032 |
| Volume of inferior parietal | -0.025 | 0.016 | -0.106 | 0.062 |
| Volume of inferior temporal | -0.014 | 0.029 | -0.043 | 0.002 |
| Volume of insula | -0.002 | 0.004 | -0.026 | 0.01 |
| Volume of isthmus cingulate | -0.054 | 0.025 | 0 | -0.041 |
| Volume of lateraloccipital | -0.021 | 0.015 | -0.062 | 0.027 |
| Volume of lateralorbitofrontal | -0.039 | 0.027 | -0.072 | 0.094 |
| Volume of lingual | -0.017 | 0.01 | -0.056 | -0.035 |
| Volume of medial orbitofrontal | -0.006 | 0.034 | -0.066 | -0.046 |
| Volume of middle temporal | -0.005 | 0.02 | 0 | 0.018 |
| Volume of paracentral | 0.012 | -0.006 | -0.006 | 0.019 |
| Volume of parahippocampal | -0.034 | -0.029 | -0.043 | -0.113 |
| Volume of parsopercularis | -0.028 | 0.001 | -0.055 | -0.029 |
| Volume of parsorbitalis | -0.025 | 0.011 | -0.018 | 0.019 |
| Volume of parstriangularis | 0.001 | -0.008 | -0.003 | 0.011 |
| Volume of pericalcarine | -0.015 | 0.017 | -0.029 | -0.057 |
| Volume of postcentral | -0.009 | 0.021 | -0.019 | 0.117 |
| Volume of posterior cingulate | -0.015 | 0.005 | 0.015 | 0.023 |
| Volume of precentral | -0.008 | 0.013 | -0.017 | 0.091 |
| Volume of precuneus | -0.02 | 0.009 | -0.048 | -0.018 |
| Volume of rostralanterior cingulate | 0.016 | 0.022 | 0.042 | -0.042 |
| Volume of rostralmiddle frontal | 0.004 | 0.061 | -0.049 | 0.026 |
| Volume of superior frontal | -0.008 | 0.023 | -0.054 | 0.014 |
| Volume of superior parietal | -0.018 | 0.01 | -0.071 | -0.139 |
| Volume of superior temporal | -0.013 | -0.004 | -0.073 | -0.057 |
| Volume of supramarginal | -0.017 | -0.032 | -0.064 | 0.037 |
| Volume of transversetemporal | -0.023 | -0.01 | -0.04 | 0.029 |

***S3.6.4 Subcortical Measures***

Effect sizes for the association of the interaction of mental health traits and seasonality with subcortical brain imaging measures. P-RMDD., Probable Recurrent Major Depressive Disorder; P-SEMDD., Probable Single episode Major Depressive Disorder; P-BD., Probable Bipolar Depression; P-UM., Probable Unipolar Mania. * represents FDR corrected p-value <0.05.

| **Model** | **P-RMDD** | **P-SEMDD** | **P-BD** | **P-UM** |
| --- | --- | --- | --- | --- |
| Volume Of accumbens | -0.009 | -0.018 | -0.037 | 0.038 |
| Volume Of amygdala | -0.009 | -0.011 | -0.137* | 0.089 |
| Volume Of caudate | -0.031 | 0.069 | -0.052 | -0.024 |
| Volume Of hippocampus | -0.044 | 0.006 | -0.088 | -0.148 |
| Volume Of pallidum | -0.013 | 0.03 | -0.08 | 0.003 |
| Volume Of putamen | -0.006 | 0.04 | -0.014 | -0.028 |
| Volume Of thalamus | -0.042 | 0.026 | -0.076 | 0.004 |

***S3.6.5 DTI Global Measures***

Effect sizes for the association of the interaction of mental health traits and seasonality with global DTI brain imaging measures. P-RMDD., Probable Recurrent Major Depressive Disorder; P-SEMDD., Probable Single episode Major Depressive Disorder; P-BD., Probable Bipolar Depression; P-UM., Probable Unipolar Mania. * represents FDR corrected p-value <0.05.

| **Model** | **P-RMDD** | **P-SEMDD** | **P-BD** | **P-UM** |
| --- | --- | --- | --- | --- |
| FA Total Tracts | 0.009 | -0.026 | -0.092 | 0.067 |
| MD Total Tracts | -0.026 | 0.04 | 0.059 | -0.044 |

***S3.6.6 DTI Grouped Tract Measures***

Effect sizes for the association of the interaction of mental health traits and seasonality with DTI grouped tract brain imaging measures. P-RMDD., Probable Recurrent Major Depressive Disorder; P-SEMDD., Probable Single episode Major Depressive Disorder; P-BD., Probable Bipolar Depression; P-UM., Probable Unipolar Mania. * represents FDR corrected p-value <0.05.

| **Model** | **P-RMDD** | **P-SEMDD** | **P-BD** | **P-UM** |
| --- | --- | --- | --- | --- |
| FA Association Fibers | 0.014 | -0.016 | -0.1 | 0.058 |
| FA Projection Fibers | 0.013 | -0.006 | -0.058 | 0.049 |
| FA Thalamic Radiations | -0.006 | -0.059 | -0.088 | 0.089 |
| MD Association Fibers | -0.027 | 0.032 | 0.083 | -0.026 |
| MD Projection Fibers | -0.026 | 0.044 | 0.028 | 0.018 |
| MD Thalamic Radiations | -0.014 | 0.043 | 0.039 | -0.077 |

***S3.6.7 DTI Individual Tract Measures***

Effect sizes for the association of the interaction of mental health traits and seasonality with individual DTI brain imaging measures. P-RMDD., Probable Recurrent Major Depressive Disorder; P-SEMDD., Probable Single episode Major Depressive Disorder; P-BD., Probable Bipolar Depression; P-UM., Probable Unipolar Mania. * represents FDR corrected p-value <0.05.

| **Model** | **P-RMDD** | **P-SEMDD** | **P-BD** | **P-UM** |
| --- | --- | --- | --- | --- |
| FA cingulate gyrus part of cingulum | 0.022 | -0.015 | -0.085 | -0.019 |
| FA parahippocampal part of cingulum | 0.02 | 0.025 | -0.042 | 0.06 |
| FA inferior frontooccipital fasciculus | -0.003 | -0.025 | -0.054 | 0.074 |
| FA superior longitudinal fasciculus | -0.008 | -0.029 | -0.07 | 0.05 |
| FA uncinate fasciculus | 0 | 0.017 | -0.056 | 0.137 |
| FA inferior longitudinal fasciculus | 0.002 | -0.03 | -0.04 | -0.003 |
| FA corticospinal tract | -0.002 | 0.002 | -0.015 | 0.036 |
| FA acoustic radiation | 0.009 | -0.001 | -0.071 | -0.094 |
| FA medial lemniscus | 0.012 | -0.001 | -0.009 | 0.002 |
| FA superior thalamic radiation | -0.011 | -0.042 | -0.097 | -0.013 |
| FA posterior thalamic radiation | -0.002 | -0.055 | -0.052 | 0.059 |
| FA anterior thalamic radiation | -0.002 | -0.037 | -0.063 | 0.157 |
| MD cingulate gyrus part of cingulum | -0.023 | 0.026 | 0.049 | 0.009 |
| MD parahippocampal part of cingulum | -0.029 | 0.014 | 0.086 | -0.018 |
| MD inferior frontooccipital fasciculus | -0.009 | 0.028 | 0.04 | -0.006 |
| MD superior longitudinal fasciculus | 0.004 | 0.04 | 0.022 | -0.032 |
| MD uncinate fasciculus | -0.015 | 0.023 | 0.048 | -0.077 |
| MD inferior longitudinal fasciculus | -0.014 | 0.027 | 0.024 | 0.028 |
| MD corticospinal tract | -0.049 | 0.025 | 0.004 | -0.02 |
| MD acoustic radiation | -0.044 | 0.008 | -0.032 | -0.08 |
| MD medial lemniscus | 0.008 | 0.018 | 0.005 | -0.125 |
| MD superior thalamic radiation | -0.014 | 0.013 | 0.037 | -0.036 |
| MD posterior thalamic radiation | -0.001 | 0.041 | 0.026 | -0.038 |
| MD anterior thalamic radiation | -0.026 | 0.042 | 0.043 | -0.125 |
| FA middle cerebellar peduncle | 0.009 | 0.025 | -0.051 | 0.185 |
| FA forceps major | 0.014 | -0.027 | -0.081 | 0.058 |
| FA forceps minor | 0.012 | -0.048 | 0.023 | 0.044 |
| MD middle cerebellar peduncle | -0.013 | 0.038 | 0.021 | 0.111 |
| MD forceps major | -0.009 | 0.019 | 0.085 | 0.027 |
| MD forceps minor | -0.019 | 0.01 | -0.029 | -0.063 |

***S3.7 Seasonality associations with brain imaging measures when covarying for mental health traits and the interaction between mental health traits and seasonality***

***S3.7.1 Global T1 measures***

Effect sizes for seasonality associations with global T1 brain imaging measures. P-RMDD., Probable Recurrent Major Depressive Disorder; P-SEMDD., Probable Single episode Major Depressive Disorder; P-BD., Probable Bipolar Depression; P-UM., Probable Unipolar Mania. * represents FDR corrected p-value <0.05.

| **Model** | **P-RMDD** | **P-SEMDD** | **P-BD** | **P-UM** |
| --- | --- | --- | --- | --- |
| Global Surface Area | 0.003 | 0.003 | 0.003 | 0.003 |
| Global Cortical Volume | 0 | 0 | 0 | 0.001 |
| Global Cortical Thickness | 0.007 | 0.007 | 0.007 | 0.007 |

***S3.7.2 Lobar T1 measures***
Effect sizes for seasonality associations with lobar T1 brain imaging measures. P-RMDD., Probable Recurrent Major Depressive Disorder; P-SEMDD., Probable Single episode Major Depressive Disorder; P-BD., Probable Bipolar Depression; P-UM., Probable Unipolar Mania. * represents FDR corrected p-value <0.05.

| **Model** | **P-RMDD** | **P-SEMDD** | **P-BD** | **P-UM** |
| --- | --- | --- | --- | --- |
| Frontal Area | -0.005 | -0.005 | -0.005 | -0.005 |
| Frontal Thickness | -0.006 | -0.006 | -0.006 | -0.006 |
| Frontal Volume | -0.009 | -0.009 | -0.009 | -0.009 |
| Temporal Area | 0.008 | 0.009 | 0.009 | 0.009 |
| Temporal Thickness | 0.016 | 0.017 | 0.017 | 0.017 |
| Temporal Volume | 0.011 | 0.012 | 0.012 | 0.012 |
| Cingulate Area | -0.005 | -0.005 | -0.005 | -0.005 |
| Cingulate Thickness | 0.009 | 0.009 | 0.009 | 0.009 |
| Cingulate Volume | -0.006 | -0.006 | -0.005 | -0.005 |
| Parietal Area | 0.009 | 0.009 | 0.009 | 0.009 |
| Parietal Thickness | -0.009 | -0.008 | -0.008 | -0.009 |
| Parietal Volume | 0.003 | 0.003 | 0.003 | 0.003 |
| Occipital Area | 0.003 | 0.003 | 0.003 | 0.003 |
| Occipital Thickness | 0.002 | 0.002 | 0.002 | 0.002 |
| Occipital Volume | -0.001 | -0.001 | -0.001 | -0.001 |

***S3.7.3 Individual T1 measures***

Effect sizes for seasonality associations with individual T1 brain imaging measures. P-RMDD. Probable Recurrent Major Depressive Disorder; P-SEMDD., Probable Single episode Major Depressive Disorder; P-BD., Probable Bipolar Depression; P-UM., Probable Unipolar Mania. * represents FDR corrected p-value <0.05.

| **Model** | **P-RMDD** | **P-SEMDD** | **P-BD** | **P-UM** |
| --- | --- | --- | --- | --- |
| Area of caudal anterior cingulate | -0.014 | -0.014 | -0.014 | -0.014 |
| Area of caudal middle frontal | -0.004 | -0.003 | -0.003 | -0.003 |
| Area of cuneus | -0.019 | -0.019 | -0.019 | -0.019 |
| Area of entorhinal | 0.029 | 0.029 | 0.029 | 0.029 |
| Area of fusiform | 0.014 | 0.014 | 0.014 | 0.015 |
| Area of inferior parietal | 0.019 | 0.02 | 0.019 | 0.02 |
| Area of inferior temporal | 0.007 | 0.007 | 0.007 | 0.007 |
| Area of insula | -0.006 | -0.006 | -0.006 | -0.005 |
| Area of isthmus cingulate | 0.013 | 0.013 | 0.013 | 0.013 |
| Area of lateraloccipital | 0.009 | 0.009 | 0.009 | 0.009 |
| Area of lateralorbitofrontal | 0.004 | 0.004 | 0.004 | 0.004 |
| Area of lingual | 0.01 | 0.01 | 0.01 | 0.01 |
| Area of medial orbitofrontal | -0.01 | -0.01 | -0.01 | -0.01 |
| Area of middle temporal | 0.009 | 0.009 | 0.009 | 0.009 |
| Area of paracentral | -0.006 | -0.005 | -0.005 | -0.005 |
| Area of parahippocampal | -0.004 | -0.004 | -0.004 | -0.004 |
| Area of parsopercularis | 0.023 | 0.023 | 0.023 | 0.023 |
| Area of parsorbitalis | -0.004 | -0.004 | -0.004 | -0.003 |
| Area of parstriangularis | 0.005 | 0.005 | 0.005 | 0.005 |
| Area of pericalcarine | -0.004 | -0.004 | -0.004 | -0.004 |
| Area of postcentral | -0.001 | -0.001 | -0.001 | -0.001 |
| Area of posterior cingulate | -0.003 | -0.003 | -0.003 | -0.002 |
| Area of precentral | -0.002 | -0.002 | -0.002 | -0.002 |
| Area of precuneus | 0.007 | 0.007 | 0.007 | 0.007 |
| Area of rostralanterior cingulate | -0.004 | -0.004 | -0.004 | -0.004 |
| Area of rostralmiddle frontal | -0.012 | -0.012 | -0.012 | -0.012 |
| Area of superior frontal | -0.009 | -0.009 | -0.009 | -0.009 |
| Area of superior parietal | 0.005 | 0.005 | 0.005 | 0.005 |
| Area of superior temporal | 0.004 | 0.004 | 0.004 | 0.004 |
| Area of supramarginal | 0.003 | 0.003 | 0.003 | 0.003 |
| Area of transversetemporal | -0.006 | -0.005 | -0.005 | -0.005 |
| Volume of caudal anterior cingulate | -0.007 | -0.006 | -0.006 | -0.006 |
| Volume of caudal middle frontal | -0.007 | -0.006 | -0.006 | -0.006 |
| Volume of cuneus | -0.01 | -0.01 | -0.01 | -0.01 |
| Volume of entorhinal | 0.025 | 0.025 | 0.025 | 0.025 |
| Volume of fusiform | 0.018 | 0.018 | 0.018 | 0.018 |
| Volume of inferior parietal | 0.016 | 0.016 | 0.016 | 0.016 |
| Volume of inferior temporal | 0.005 | 0.006 | 0.006 | 0.006 |
| Volume of insula | -0.007 | -0.007 | -0.007 | -0.007 |
| Volume of isthmus cingulate | 0.017 | 0.017 | 0.017 | 0.017 |
| Volume of lateraloccipital | 0.003 | 0.003 | 0.003 | 0.003 |
| Volume of lateralorbitofrontal | 0.004 | 0.004 | 0.004 | 0.004 |
| Volume of lingual | 0.003 | 0.003 | 0.003 | 0.003 |
| Volume of medial orbitofrontal | -0.011 | -0.011 | -0.011 | -0.011 |
| Volume of middle temporal | 0.008 | 0.008 | 0.008 | 0.008 |
| Volume of paracentral | -0.009 | -0.009 | -0.009 | -0.009 |
| Volume of parahippocampal | 0.015 | 0.015 | 0.015 | 0.015 |
| Volume of parsopercularis | 0.02 | 0.02 | 0.02 | 0.02 |
| Volume of parsorbitalis | 0.005 | 0.005 | 0.005 | 0.006 |
| Volume of parstriangularis | 0.001 | 0.001 | 0.001 | 0.001 |
| Volume of pericalcarine | -0.007 | -0.007 | -0.007 | -0.007 |
| Volume of postcentral | -0.008 | -0.008 | -0.008 | -0.008 |
| Volume of posterior cingulate | -0.008 | -0.008 | -0.008 | -0.007 |
| Volume of precentral | -0.007 | -0.006 | -0.006 | -0.006 |
| Volume of precuneus | 0 | 0 | 0 | 0 |
| Volume of rostralanterior cingulate | -0.008 | -0.009 | -0.008 | -0.008 |
| Volume of rostralmiddle frontal | -0.014 | -0.014 | -0.014 | -0.014 |
| Volume of superior frontal | -0.013 | -0.013 | -0.013 | -0.013 |
| Volume of superior parietal | -0.002 | -0.002 | -0.002 | -0.002 |
| Volume of superior temporal | 0.009 | 0.009 | 0.009 | 0.009 |
| Volume of supramarginal | 0.001 | 0.001 | 0.001 | 0.001 |
| Volume of transversetemporal | -0.002 | -0.002 | -0.002 | -0.002 |

***S3.7.4 Subcortical Measures***

Effect sizes for seasonality associations with subcortical brain imaging measures. P-RMDD., Probable Recurrent Major Depressive Disorder; P-SEMDD., Probable Single episode Major Depressive Disorder; P-BD., Probable Bipolar Depression; P-UM., Probable Unipolar Mania. * represents FDR corrected p-value <0.05.

| **Model** | **P-RMDD** | **P-SEMDD** | **P-BD** | **P-UM** |
| --- | --- | --- | --- | --- |
| Volume Of accumbens | 0.006 | 0.005 | 0.006 | 0.006 |
| Volume Of amygdala | 0.021 | 0.021 | 0.021 | 0.021 |
| Volume Of caudate | 0.009 | 0.009 | 0.009 | 0.009 |
| Volume Of hippocampus | 0.019 | 0.019 | 0.019 | 0.019 |
| Volume Of pallidum | 0.007 | 0.007 | 0.007 | 0.007 |
| Volume Of putamen | 0.005 | 0.005 | 0.005 | 0.005 |
| Volume Of thalamus | 0.008 | 0.008 | 0.008 | 0.009 |

***S3.7.5 DTI Global Measures***

Effect sizes for seasonality associations with global DTI brain imaging measures. P-RMDD., Probable Recurrent Major Depressive Disorder; P-SEMDD., Probable Single episode Major Depressive Disorder; P-BD., Probable Bipolar Depression; P-UM., Probable Unipolar Mania. * represents FDR corrected p-value <0.05.

| **Model** | **P-RMDD** | **P-SEMDD** | **P-BD** | **P-UM** |
| --- | --- | --- | --- | --- |
| FA Total Tracts | -0.016 | -0.016 | -0.016 | -0.016 |
| MD Total Tracts | -0.002 | -0.002 | -0.002 | -0.002 |

***S3.7.6 DTI Grouped Tract Measures***

Effect sizes for seasonality associations with DTI grouped tract measures. P-RMDD., Probable Recurrent Major Depressive Disorder; P-SEMDD., Probable Single episode Major Depressive Disorder; P-BD., Probable Bipolar Depression; P-UM., Probable Unipolar Mania. * represents FDR corrected p-value <0.05.

| **Model** | **P-RMDD** | **P-SEMDD** | **P-BD** | **P-UM** |
| --- | --- | --- | --- | --- |
| FA Association Fibers | -0.024 | -0.024 | -0.024 | -0.024 |
| FA Projection Fibers | -0.002 | -0.002 | -0.002 | -0.002 |
| FA Thalamic Radiations | -0.009 | -0.009 | -0.009 | -0.009 |
| MD Association Fibers | -0.001 | -0.001 | -0.001 | -0.001 |
| MD Projection Fibers | 0.002 | 0.002 | 0.002 | 0.002 |
| MD Thalamic Radiations | -0.001 | -0.001 | -0.002 | -0.002 |

***S3.7.7 DTI Individual Tract Measures***

Effect sizes for seasonality associations with individual DTI brain imaging measures. P-RMDD., Probable Recurrent Major Depressive Disorder; P-SEMDD., Probable Single episode Major Depressive Disorder; P-BD., Probable Bipolar Depression; P-UM., Probable Unipolar Mania. * represents FDR corrected p-value <0.05.

| **Model** | **P-RMDD** | **P-SEMDD** | **P-BD** | **P-UM** |
| --- | --- | --- | --- | --- |
| FA cingulate gyrus part of cingulum | -0.022 | -0.022 | -0.022 | -0.021 |
| FA parahippocampal part of cingulum | -0.025 | -0.024 | -0.024 | -0.024 |
| FA inferior frontooccipital fasciculus | -0.021 | -0.021 | -0.021 | -0.021 |
| FA superior longitudinal fasciculus | 0.005 | 0.005 | 0.006 | 0.006 |
| FA uncinate fasciculus | -0.004 | -0.004 | -0.004 | -0.004 |
| FA inferior longitudinal fasciculus | -0.014 | -0.014 | -0.014 | -0.014 |
| FA corticospinal tract | -0.001 | -0.001 | -0.001 | -0.001 |
| FA acoustic radiation | -0.005 | -0.005 | -0.005 | -0.005 |
| FA medial lemniscus | 0.015 | 0.016 | 0.016 | 0.016 |
| FA superior thalamic radiation | 0.015 | 0.015 | 0.015 | 0.015 |
| FA posterior thalamic radiation | -0.017 | -0.016 | -0.016 | -0.016 |
| FA anterior thalamic radiation | -0.015 | -0.015 | -0.015 | -0.015 |
| MD cingulate gyrus part of cingulum | 0.003 | 0.002 | 0.002 | 0.002 |
| MD parahippocampal part of cingulum | 0.008 | 0.008 | 0.008 | 0.008 |
| MD inferior frontooccipital fasciculus | -0.009 | -0.009 | -0.009 | -0.009 |
| MD superior longitudinal fasciculus | -0.011 | -0.011 | -0.011 | -0.011 |
| MD uncinate fasciculus | -0.006 | -0.006 | -0.006 | -0.006 |
| MD inferior longitudinal fasciculus | -0.009 | -0.009 | -0.009 | -0.009 |
| MD corticospinal tract | 0.011 | 0.01 | 0.01 | 0.01 |
| MD acoustic radiation | 0.003 | 0.003 | 0.002 | 0.002 |
| MD medial lemniscus | 0.005 | 0.004 | 0.005 | 0.004 |
| MD superior thalamic radiation | 0.016 | 0.016 | 0.016 | 0.016 |
| MD posterior thalamic radiation | -0.011 | -0.011 | -0.011 | -0.011 |
| MD anterior thalamic radiation | 0.002 | 0.002 | 0.002 | 0.002 |
| FA middle cerebellar peduncle | -0.012 | -0.013 | -0.012 | -0.012 |
| FA forceps major | -0.003 | -0.003 | -0.003 | -0.003 |
| FA forceps minor | -0.008 | -0.008 | -0.008 | -0.008 |
| MD middle cerebellar peduncle | 0.004 | 0.004 | 0.004 | 0.004 |
| MD forceps major | -0.015 | -0.014 | -0.015 | -0.015 |
| MD forceps minor | -0.005 | -0.005 | -0.005 | -0.005 |

***S4. Tabular summary of directions of effect for variables with significant associations for all analyses.***
Direction of effect is represented by + (positive) and - (negative) for all associations with FDR corrected p-value <0.05. S (Main)., main analysis; S (Sen.)., sensitivity analysis; P-RMDD., Probable Recurrent Major Depressive Disorder; P-SEMDD., Probable Single Episode Major Depressive Disorder; P-BD., Probable Bipolar Disorder; P-BD*S., interaction between Probable Bipolar Disorder and seasonality on brain imaging measures. The variables examined in the main and sensitivity analyses were seasonality.

| **Model** | **S (Main)** | **S (Sen.)** | **P-RMDD** | **P-SEMDD** | **P-MDD** | **P-BD** | **P-BD*S** |
| --- | --- | --- | --- | --- | --- | --- | --- |
| Cingulate Thickness |  | **+** |  |  |  |  |  |
| FA Association Fibers | **-** | **-** | **-** |  | **-** |  |  |
| FA anterior thalamic radiation | **-** | **-** | **-** |  | **-** |  |  |
| FA cingulate gyrus part of cingulum | **-** | **-** | **-** |  | **-** |  |  |
| FA forceps major | **-** | **-** |  |  |  |  |  |
| FA forceps minor |  | **-** | **-** |  | **-** |  |  |
| FA inferior frontooccipital fasciculus | **-** | **-** | **-** |  | **-** |  |  |
| FA inferior longitudinal fasciculus | **-** | **-** | **-** |  | **-** |  |  |
| FA posterior thalamic radiation | **-** | **-** | **-** |  | **-** | **-** |  |
| FA superior longitudinal fasciculus |  |  | **-** |  | **-** |  |  |
| FA superior thalamic radiation |  |  | **-** |  | **-** |  |  |
| FA uncinate fasciculus |  |  | **-** |  | **-** |  |  |
| FA Thalamic Radiations | **-** | **-** | **-** |  | **-** | **-** |  |
| FA Total Tracts | **-** | **-** | **-** | **-** | **-** |  |  |
| Frontal Volume |  |  |  | **-** |  |  |  |
| Global Cortical Thickness |  |  |  | **-** |  |  |  |
| Global Cortical Volume |  |  |  | **-** |  |  |  |
| Global Surface Area |  |  |  | **-** |  |  |  |
| MD Association Fibers |  |  | **+** |  | **+** |  |  |
| MD anterior thalamic radiation |  |  | **+** |  | **+** |  |  |
| MD cingulate gyrus part of cingulum |  |  | **+** |  | **+** |  |  |
| MD corticospinal tract |  |  | **+** |  | **+** |  |  |
| MD forceps minor |  |  | **+** |  | **+** |  |  |
| MD inferior frontooccipital fasciculus |  |  | **+** |  | **+** |  |  |
| MD inferior longitudinal fasciculus |  |  | **+** |  | **+** |  |  |
| MD middle cerebellar peduncle |  |  | **+** |  | **+** |  |  |
| MD posterior thalamic radiation |  |  | **+** |  | **+** |  |  |
| MD superior longitudinal fasciculus |  |  | **+** |  | **+** |  |  |
| MD superior thalamic radiation |  |  | **+** |  | **+** |  |  |
| MD uncinate fasciculus |  |  | **+** |  | **+** |  |  |
| MD Projection Fibers |  |  | **+** |  | **+** |  |  |
| MD Thalamic Radiations |  |  | **+** |  | **+** |  |  |
| MD Total Tracts |  |  | **+** | **+** | **+** |  |  |
| Occipital Thickness | **+** | **+** |  |  |  |  |  |
| Temporal Thickness | **+** | **+** |  |  |  |  |  |
| Volume Of amygdala |  | **+** |  |  |  |  | **-** |
| Volume of medial orbitofrontal |  |  |  |  | **-** |  |  |
| Volume Of thalamus |  |  | **-** |  | **-** |  |  |

***S5. Extended results of mental health trait associations with brain imaging measures***

Across global white matter microstructure measures, P-MDD showed similar associations to P-RMDD and P-SEMDD, with effect sizes closer to those observed for P-RMDD than for P-SEMDD (See S3.5.5 Results). Morphologically, only SEMDD was associated with significant reductions in all three global measures (CSA: β= -0.044, CT: β= -0.044, CV: β= -0.048, *p=<*0.05) (See S3.4.1 Results). All regional WM microstructure associations found for P-RMDD remained when P-MDD was examined (See S3.4.6 Results).

Nine white matter tracts were associated with P-RMDD after multiple testing correction (β= -0.092 to -0.033, *p_corr_*=<0.05) with lower FA in the posterior thalamic radiation exhibiting the strongest association (β= -0.092, *p_corr_*=<0.0001) (See S7.Fig, S8.Fig, S3.4.7 Results). Respective higher MD in these measures was also associated with P-RMDD, with the addition of higher MD in the corticospinal tract and inferior cerebellar peduncle (β= 0.037 to 0.078, *p_corr_*=<0.05). These results were consistent with those of P-MDD. BD showed significant associations with lower FA in posterior thalamic radiations (β= -0.12, *p_corr_*=<0.05). No significant associations were found for P-SEMDD or P-UM. Reduced CV in the medial orbitofrontal cortex was the only individual measure exclusively associated with P-MDD (β= -0.046 *p_corr_*=0.01) (See S3.5.3 Results).

There was a subcortical association between decreased thalamic volume for both P-RMDD (β= -0.044, *p_corr_*=0.028) (See S3.4.4 Results) and P-MDD (β= -0.044, *p_corr_*=0.006) (See S3.5.4 Results) as well as a negative interaction between P-BD and seasonality for amygdala SV (β= -0.137, *p_corr_*=0.01) (See S3.6.4 Results).

`No differences were observed between cases when comparing P-RMDD and P-SEMDD (See S3.5.6 Results).


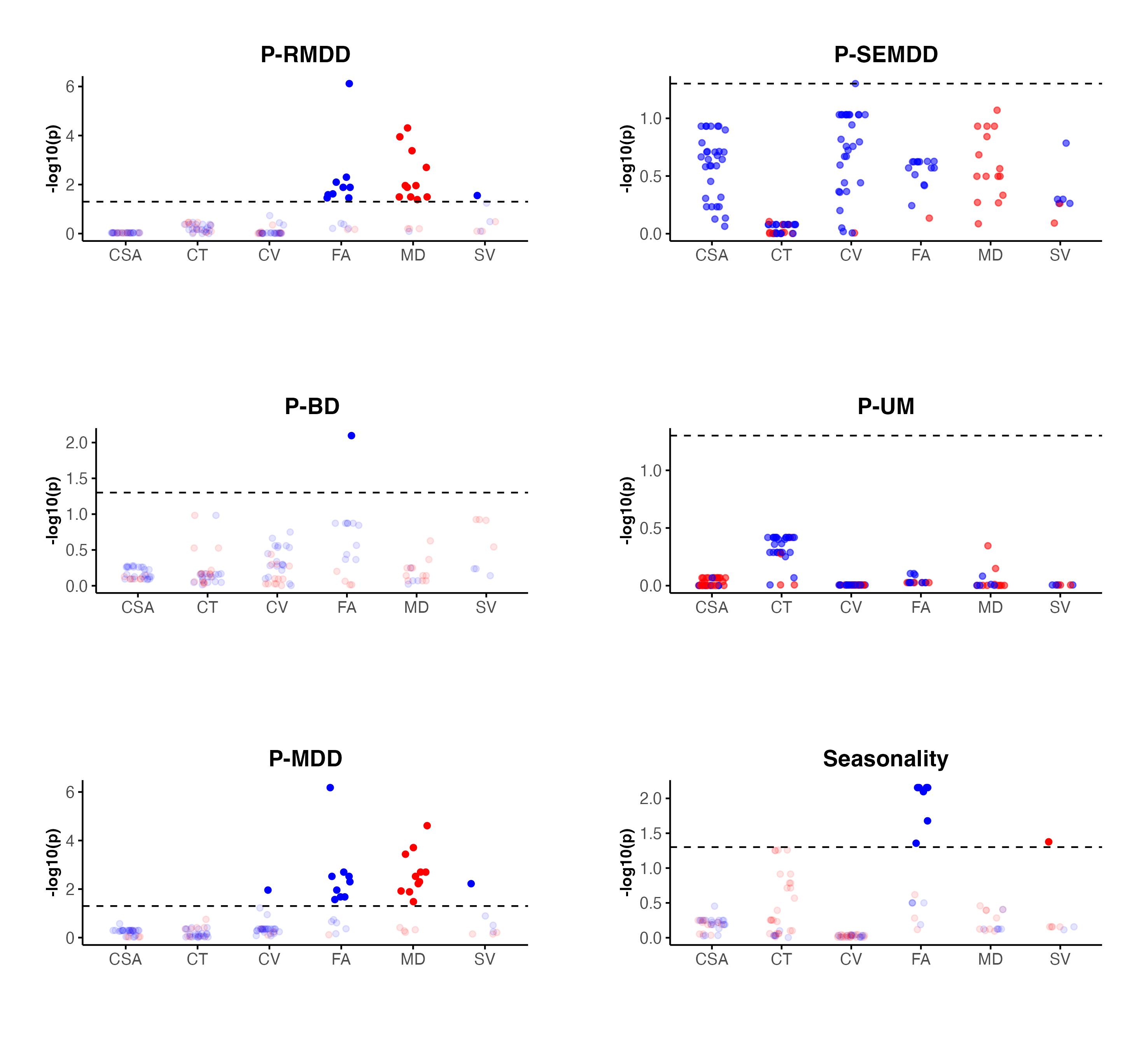
**S7. Fig.** Individual white matter microstructure and brain morphology measure associations with mental health traits and seasonality. Seasonality associations are from the sensitivity analysis. P-RMDD = Probable Recurrent Major Depressive Disorder; P-SEMDD = Probable Single episode Major Depressive Disorder; P-BD = Probable Bipolar Disorder; P-UM = Probable Unipolar Mania; P-MDD = Probable Major Depressive Disorder; CSA = cortical surface area; CV = cortical volume; SV = subcortical volume; FA = fractional anisotropy; MD = mean diffusivity. Red and blue dots represent positive and negative associations, respectively. Horizontal dashed lines represent p= 0.05; bold dots signify significant FDR corrected associations (*p_corr_*=<0.05).


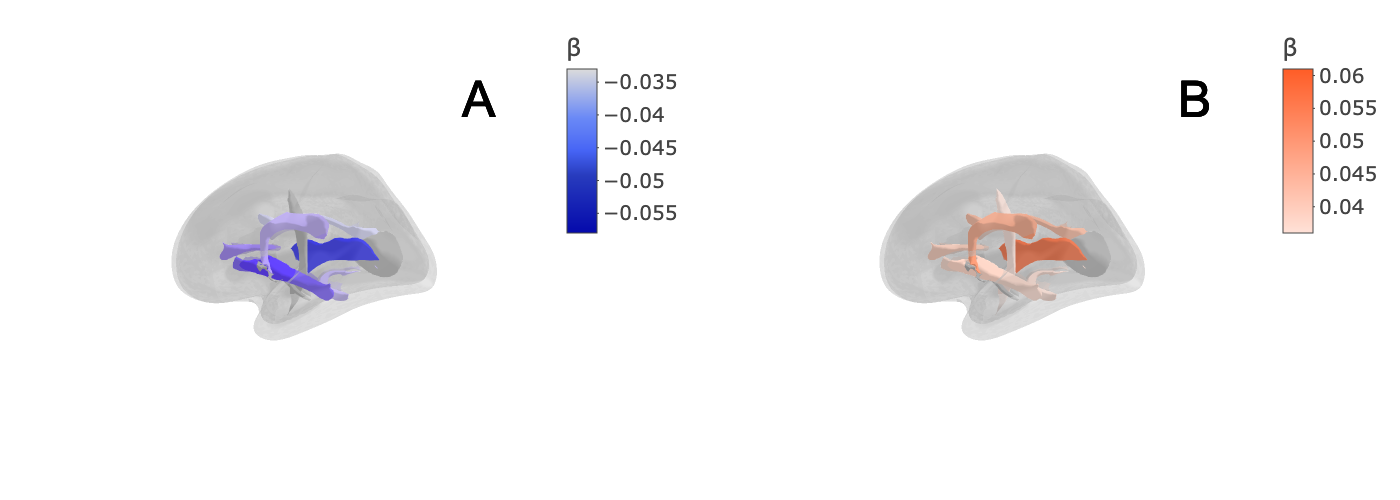
**S8. Fig.** Significant associations (*p_corr_*<0.05) between (A) Probable Recurrent Major Depressive Disorder and individual fractional anisotropy white matter microstructure measures and (B) Probable Recurrent Major Depressive Disorder and individual mean diffusivity white matter microstructure measures. Blue colour represents a decrease in fractional anisotropy measures and red colour represents an increase in mean diffusivity measures. Darker colour designates greater effect of mental health trait on the individual measure.
