# Supplementary material for: An epidemiological study of season of birth, mental health, and neuroimaging in the UK Biobank
Source: PLoS One. 2024 May 22;19(5):e0300449. doi: 10.1371/journal.pone.0300449 (PMC11111058; doi:10.1371/journal.pone.0300449)
Supplement: Attachment — Submitted filename: Response_To_Reviewers.docx [file pone.0300449.s002.docx]

**PLOS ONE**
**MS Number: PONE-D-23-25155**
**Title: An epidemiological study of season of birth, mental health, and neuroimaging in the UK Biobank**

All page numbers reported below refer to the clean version (i.e. accepted tracked changes) of the submitted manuscript. We include a tracked changes version of the manuscript in the resubmission as well to help reviewers and editors to identify those sections that have been changed and updated.

**Reviewer #1**

I have read with interest the manuscript by Viejo-Romero et al. on the effect of season of birth on mental health in adults. It is especially relevant the fact that they use a large and high-quality database as the UK Biobank to study this effect, which has received moderate attention in recent years. Authors find small effects relating probable recurrent Major Depressive Disorder with the summer. However they do not find a similar effect with probable hypomania, mania or, importantly, single episode Major Depressive Disorder. Additionally, they find a high number of neuroimaging correlations.
Overall, reporting is of high quality and follows current standards. However, I have some issues with the article as it stands that question the robustness of its results and if solved would probably improve the paper and its addition to the field.
I have to say that I am slightly biased against the hypothesis that authors propose, as I do not think that there is a strong biological basis for any long-last consequences of date of birth. The article does not help against my skepticism of the field. All in all, the authors are not able to convincingly proof whether there is something to be learnt from the current study or it is simply an statistical fluke.
Regarding the neuroimaging data, the relevance of its results is hard to assess.

**General response to Reviewer #1**

We wish to thank Reviewer #1 for their insightful comments, and they raised some very important points. The manuscript is now much stronger for the contributions from this reviewer, and we hope they will agree that their comments have been comprehensively addressed.

**Reviewer #1, Comment #1**

As it is frequent in the neuroimaging literature, some correlations are found without any possibility to judge whether they are relevant or not… I cannot judge this section as one is easily lost in the jungle of measures and RoIs… I would however recommend the authors to include a paragraph on how the FDR correction holds when it is applied to many different comparisons independently.

**Response to Reviewer #1, Comment #1**

We thank the reviewer for this comment. We have now cited the literature within the FDR correction section of the Methods to provide examples of how this FDR method for multiple correction is frequently applied in this type of study design, lines 201-202 and below:

“False Discovery Rate (FDR) correction was applied separately across individual and regional brain morphology and white matter microstructure measures per modality (Methods S2.4.1) (Shen et al., 2017; Harris et al., 2022).”

**Reviewer #1, Comment #2**

The models directly linking date of birth and risk of mental health issues are slightly more interpretable. However, they are far from being straightforward. Indeed the phenotypic score for season (“The birth month of each participant was transformed via a cos function, with the lowest phenotypic score (-1) corresponding to those born in December and the highest phenotypic score corresponding to those born in June (+1)”) is only one of multiple parametrizations, which is selected without justification.

**Response to Reviewer #1, Comment #2**

The phenotypic score was parametrized in this way following Howard et al., 2021 (1), to capture differences between winter and summer births. Similar studies in UKB have achieved this by phenotyping participants according to day length (short: October-March, long: April-Sept) (2), and winter/spring and summer/autumn(3). There is little consensus, therefore, on how seasonality of birth is defined, and the limitations of this phenotype are presently acknowledged within the manuscript in lines 336-340 and below:

“Here, birth seasonality variation was assessed comparing winter births with summer births. However, this provides those born in spring and autumn with similar phenotypic scores and therefore will not model differences between those born in those periods. Future studies could co-model a range of seasonality phenotypes to contextualise the extent of these potential differences.”.

**Reviewer #1, Comment #3**

Even more importantly, associations between the four mental health phenotypes and seasonality were tested using a complex logistic binomial regression analysis in which sex, age, age2, Townsend Deprivation Index, assessment centre attended and place of birth location were included as covariates. There is no description on how authors arrived at the selection of these factors. My suspicion is that the result found may not be robust to changes in the proposed model and may be the result of the “garden of forking paths” and the researcher’s degrees of freedom in their design. The following actions could provide additional confidence in the results: **(1)** Proof indicating that these choices were carried out a-priori (eg: a public protocol or previous references by the authors or other researchers using the exact same parametrizations and models). If not possible, at least some information on how and why they were selected. **(2)** A declaration by the authors indicating that this was the only model tested. **(3)** Some sort of multiverse testing or robustness checks showing how different changes in the analytical decisions affect the results. An easy one would be to show the results of the logistic model with different combinations of the covariates.

**Response to Reviewer #1, Comment #3 (1)**

We thank the reviewer for their comments. We understand the importance of justifying the a priori rationale and robustness of statistical models and have therefore provided this rationale within the manuscript (lines 172-178, 194-197) and below. In short, sex, age, and assessment centre attended are standard covariates applied throughout the majority of epidemiological studies in the UK Biobank cohort and others of this kind. Additionally, sociodemographic factors (captured by Townsend Deprivation Index (TDI) in this study), are usually employed in models testing associations between both mental health and brain biomarkers (4,5), specifically in UK Biobank given the high genetic correlations between TDI and psychiatric disorders (6,7) and associations between TDI and neuroimaging measures (8) both observed in this cohort. The inclusion of place of birth follows an acknowledgement of the association between genetic variants and birth location within UKB (9), a latent structure which the authors determined should be controlled for here as in the literature (10), given the heritability of neuroimaging measures and mental health traits.

Additionally, higher latitude births have been associated with significant reductions in lifetime risk for depression (11), and so the authors considered that controlling for latitude and longitude to some degree would be beneficial. Lastly, the inclusion of age^2^ was based upon the non-linear relationship between brain morphology/white matter microstructure measures and age, which is often controlled for by the addition of age^2^, including in UKB (12). The rationale for preserving this covariate within the mental health trait models followed similar logic in attempting to account for the non-linear relationship between age and mental health symptoms, namely for depressive symptoms (13) and especially when modelling early-life events (14).

**Response to Reviewer #1, Comment #3 (2)**

We have now added a declaration to this effect within the manuscript in lines 181 and 206-207 as per below:

“No other statistical models were tested in this study.”.

**Response to Reviewer #1, Comment #3 (3)**

We thank the reviewer for highlighting he importance of robust statistical models and therefore have now applied a range of models testing the association between the four mental health disorders and seasonality of birth separately for a range of covariates. As per the table below (which is included as Supplementary Table S3.3), we tested a minimal model with the standard epidemiological study covariates of age, sex and assessment centre attended. We then tested the additional original covariates (birth location, Townsend Deprivation Index, Age^2^), by adding them one by one into additional models. Please note, these analyses were conducted within the original sample but now including all ethnicities eligible for our analysis in UKB as per Reviewer 2’s recommendation. In brief, the significant association for seasonality with recurrent MDD remained consistent across all four models after Bonferroni multiple testing correction (p_Bon_ range: 0.028-0.042). The multiple correction procedure was applied in the same manner as in the original analysis for consistency (ie. applied over the four mental health disorders per covariate set). Additionally, effect sizes for these associations remained consistent for R-MDD (β range: 0.024-0.026) with the original analysis including only participants of white ethnicity (β=0.024), and with the same model including participants of all ethnicities (β=0.028). Due to this, and given the rationale for each of the covariates chosen above, the authors have not modified the original model used in the revised manuscript. This table has now been added to the supplementary materials (S2.4.1 Multiple testing corrections applied per neuroimaging measure modality) and is referred to in the manuscript in lines 181-187 and below:

“Post-hoc analysis was conducted to test the robustness of this model by reducing the number of covariates to sex, age and assessment centre attended and sequentially adding birth location, TDI and age2 as covariates to further binomial logistic regressions per mental health trait (n=16) (ie., covariates = age, sex, assessment centre; age, sex, assessment centre, birth location; age, sex, assessment centre, birth location, TDI; age, sex, assessment centre, birth location, TDI, age2). A Bonferroni multiple analysis correction was applied consistent with that applied in the main study.”.

| **Mental Health Trait** | **Covariates** | **Effect Size(β) / Log(OR)** | **S.E.** | **p-uncorr** | **p-corr** |
| --- | --- | --- | --- | --- | --- |
| **Recurrent Depression** | **Phenotype+sex+Age+centre** | **0.025** | **0.01** | **0.01** | **0.038** |
| **Recurrent Depression** | **Phenotype+sex+Age+centre+BirthLocation** | **0.024** | **0.01** | **0.01** | **0.042** |
| **Recurrent Depression** | **Phenotype+sex+Age+centre+BirthLocation+Townsend** | **0.026** | **0.01** | **0.007** | **0.028** |
| **Recurrent Depression** | **Phenotype+sex+Age+centre+BirthLocation+Townsend+Age2** | **0.026** | **0.01** | **0.007** | **0.028** |
| Single Depression | Phenotype+sex+Age+centre | 0.016 | 0.013 | 0.239 | 0.956 |
| Single Depression | Phenotype+sex+Age+centre+BirthLocation | 0.016 | 0.013 | 0.243 | 0.971 |
| Single Depression | Phenotype+sex+Age+centre+BirthLocation+Townsend | 0.016 | 0.013 | 0.218 | 0.874 |
| Single Depression | Phenotype+sex+Age+centre+BirthLocation+Townsend+Age2 | 0.017 | 0.013 | 0.212 | 0.847 |
| Unipolar Mania | Phenotype+sex+Age+centre | 0.064 | 0.041 | 0.12 | 0.479 |
| Unipolar Mania | Phenotype+sex+Age+centre+BirthLocation | 0.065 | 0.041 | 0.115 | 0.458 |
| Unipolar Mania | Phenotype+sex+Age+centre+BirthLocation+Townsend | 0.065 | 0.041 | 0.11 | 0.44 |
| Unipolar Mania | Phenotype+sex+Age+centre+BirthLocation+Townsend+Age2 | 0.066 | 0.041 | 0.108 | 0.432 |
| Bipolar Depression | Phenotype+sex+Age+centre | 0.006 | 0.02 | 0.762 | 1 |
| Bipolar Depression | Phenotype+sex+Age+centre+BirthLocation | 0.006 | 0.02 | 0.76 | 1 |
| Bipolar Depression | Phenotype+sex+Age+centre+BirthLocation+Townsend | 0.009 | 0.02 | 0.673 | 1 |
| Bipolar Depression | Phenotype+sex+Age+centre+BirthLocation+Townsend+Age2 | 0.009 | 0.02 | 0.665 | 1 |

**S3.3.** Mental health traits associated with seasonality under four sets of logistic regression models testing a total of four covariate sets per mental health trait. p-uncorr = p-uncorrected value; p-corr = Bonferroni p-corrected value; S.E = standard error.

**Reviewer #1, Comment #4**

In addition to the above, the article as written gives too little weight to the uncertainty present in the results. Some things that could be improved: **(1)** include in abstract the negative results with the other disorders. If authors need to cut some parts, the imaging info in the abstract could be mostly dropped. **(2)** Uncertainty of the results should be included, for example in P13-L246, but also other parts of the discussion. **(3)** Reduce Post-hoc reasoning on why negative results were found: reasons provided for example in p14 l 262 are probably less likely than the elephant in the room (that is, that the positive results found was simply an statistical fluke). **(4)** Include a section on limitations of the current study, which should include the fact that there is not a good explanation on why results where only found for recurrent MDD.

**Response to Reviewer #1, Comment #4 (1)**

We thank the reviewer for drawing attention to dealing with uncertainty in the results. We have now included the negative results for mental health and Seasonality associations within the Abstract and below:

“Due to the small effect sizes observed throughout, as well as the lack of associations with other relevant mental health traits, further research is required to validate seasonality of birth effects both in the context of different latitudes, and by co-examining genetic and epigenetic measures to potentially reveal informative biological pathways.”.

**Response to Reviewer #1, Comment #4 (2)**

We have now supplemented the Discussion with comments on the overall uncertainty and possible multiple interpretations of our results within lines 254-257 and below:

“These results cautiously lend additional support to existing evidence of season of birth effects in adult health in the context of a modest sized cross-sectional sample of a healthy UK-based population.”.

**Response to Reviewer #1, Comment #4 (3)**

Having provided evidence above of the modestly robust associations between RMDD and seasonality within this sample, we believe there is value in retaining varied discussion points on the biologically plausible mechanisms that could support this association despite the lack of association for SEMDD. However, we understand Reviewer 1’s concern and have prefaced this discussion with the following lines 279-282 and below:

“Ultimately, the discrepancy of findings for seasonality associations with recurrent and single episode MDD could be better addressed within a clinical sample, given factors such as the recall bias involved in enumerating lifetime depressive episode measures within UK Biobank (Davis et al., 2020), and the overall reliance on self-reported measures to distinguish these diagnoses.”.

**Response to Reviewer #1, Comment #4 (4)**

We thank the reviewer for this comment. We have now more clearly demarcated the existing Limitations section within the original manuscript by titling it as such (line 324). We have also reiterated the possible limitations of our methods reflected by the finding of only a single mental health association (RMDD) given within the “Mental Health Disorders” Discussion section as per the above comment and within the closing lines of the conclusion in lines 360-364 and as per below:

“Although the reported association between seasonality and recurrent Major Depressive Disorder could be specific to the parametrizations of this study and those of UK Biobank cohort, we are hopeful that they may spur further research. A continuation of the examination of seasonality associations with a wide range of mental health traits is encouraged within higher powered studies or those utilising clinical cases.”.

**Reviewer #2**

UK Biobank data are used to test the hypothesis that seasonality influences the incidence of affective disorders and brain structural measures derived from MRI. An excess number of diagnoses of recurrent depression is found during the summer months and seasonality is associated with some neuroimaging markers.

**General response to Reviewer #2**

We wish to thank Reviewer #2 for taking their time to read and review our manuscript and thank them for raising a very important issue. The manuscript is now more robust for the recommendation from this reviewer, and we hope they will agree that their comment has been comprehensively addressed.

**Reviewer #2, Comment #1**

Whilst the motivation for the conception and design is broadly sound, the exclusion of non-white participants without any scientific justification, or indeed any justification at all, is unacceptable. Whilst I am in no doubt that the authors hold no ill intentions, funding agencies – including the Wellcome Trust – are absolutely clear that scientific studies should be for the benefit of everyone. Moreover, we are ethically compelled to undertake research for the whole community unless there are specific reasons not to do so. This is particularly relevant here given that in the UK people with Black ethnicity have almost twice the prevalence of depression compared to European (white) ethnicity [doi: 10.1017/S0033291714002967]. The article requires careful reconsideration. The exclusion of any group based on their ethnicity needs strong and credible justification.

**Response to Reviewer #2, Comment #1**

We thank the reviewer for holding the authors accountable for upholding scientific ethical standards and have now adapted the study to include all ethnicities within the UK Biobank throughout all analyses presented in the manuscript. Originally the authors had restricted the ethnicities included in the sample to allow for a polygenic risk score analysis on the mental health traits and neuroimaging measures, but this was not carried out.

**References**

1. Howard DM, Pain O, Arathimos R, Barbu MC, Amador C, Walker RM, *et al.* (2022): Methylome-wide association study of early life stressors and adult mental health. *Hum Mol Genet* 31: 651–664.

2. Didikoglu A, Canal MM, Pendleton N, Payton A (2020): Seasonality and season of birth effect in the UK Biobank cohort. *American Journal of Human Biology* 32: e23417.

3. Escott-Price V, Smith DJ, Kendall K, Ward J, Kirov G, Owen MJ, *et al.* (2019): Polygenic risk for schizophrenia and season of birth within the UK Biobank cohort. *Psychological Medicine* 49: 2499–2504.

4. Topiwala A, Ebmeier KP, Maullin-Sapey T, Nichols TE (2022): Alcohol consumption and MRI markers of brain structure and function: Cohort study of 25,378 UK Biobank participants. *NeuroImage: Clinical* 35: 103066.

5. Majrashi NA, Ahearn TS, Waiter GD (2020): Brainstem volume mediates seasonal variation in depressive symptoms: A cross sectional study in the UK Biobank cohort [no. 1]. *Sci Rep* 10: 3592.

6. Hill WD, Hagenaars SP, Marioni RE, Harris SE, Liewald DCM, Davies G, *et al.* (2016): Molecular Genetic Contributions to Social Deprivation and Household Income in UK Biobank. *Current Biology* 26: 3083–3089.

7. Ye J, Wen Y, Sun X, Chu X, Li P, Cheng B, *et al.* (2021): Socioeconomic Deprivation Index Is Associated With Psychiatric Disorders: An Observational and Genome-wide Gene-by-Environment Interaction Analysis in the UK Biobank Cohort. *Biological Psychiatry* 89: 888–895.

8. Tan CH, Tan JJX (2023): Low neighborhood deprivation buffers against hippocampal neurodegeneration, white matter hyperintensities, and poorer cognition. *GeroScience* 45: 2027–2036.

9. Haworth S, Mitchell R, Corbin L, Wade KH, Dudding T, Budu-Aggrey A, *et al.* (2019): Apparent latent structure within the UK Biobank sample has implications for epidemiological analysis [no. 1]. *Nat Commun* 10: 333.

10. King C, Mulugeta A, Nabi F, Walton R, Zhou A, Hyppönen E (2020): Mendelian randomization case-control PheWAS in UK Biobank shows evidence of causality for smoking intensity in 28 distinct clinical conditions. *eClinicalMedicine* 26. https://doi.org/10.1016/j.eclinm.2020.100488

11. Devore EE, Chang S-C, Okereke OI, McMahon DG, Schernhammer ES (2018): Photoperiod during maternal pregnancy and lifetime depression in offspring. *Journal of Psychiatric Research* 104: 169–175.

12. Shen X, Reus LM, Cox SR, Adams MJ, Liewald DC, Bastin ME, *et al.* (2017): Subcortical volume and white matter integrity abnormalities in major depressive disorder: findings from UK Biobank imaging data [no. 1]. *Sci Rep* 7: 5547.

13. Airaksinen J, Komulainen K, García-Velázquez R, Määttänen I, Gluschkoff K, Savelieva K, Jokela M (2021): Subclinical hypothyroidism and symptoms of depression: Evidence from the National Health and Nutrition Examination Surveys (NHANES). *Comprehensive Psychiatry* 109: 152253.

14. Lehto K, Hägg S, Lu D, Karlsson R, Pedersen NL, Mosing MA (2020): Childhood Adoption and Mental Health in Adulthood: The Role of Gene-Environment Correlations and Interactions in the UK Biobank. *Biological Psychiatry* 87: 708–716.
