# Supplementary material for: An epidemiological study of season of birth, mental health, and neuroimaging in the UK Biobank
Source: PLoS One. 2024 May 22;19(5):e0300449. doi: 10.1371/journal.pone.0300449 (PMC11111058; doi:10.1371/journal.pone.0300449)
Supplement: Attachment — Submitted filename: Response_To_Reviewers.docx [file pone.0300449.s003.docx]

**PLOS ONE**
**MS Number: PONE-D-23-25155**
**Title: An epidemiological study of season of birth, mental health, and neuroimaging in the UK Biobank**

**Dear Editor,**

We thank you once again for the opportunity provided to re-submit our manuscript entitled “An epidemiological study of season of birth, mental health, and neuroimaging in the UK Biobank” (PONE-D-23-25155) to PLOS ONE. We provide further answers to Reviewers 2’s comments below.

**Reviewer #1: While I am still biased against the study results, authors have shown an extreme responsiveness to my comments and have provided as much evidence of the reliability of their results as it was feasible to ask. Hence, I believe that the article in its current form has greatly improved and is suitable for publication. I want to congratulate authors for their effort in their updated version.**

**Reviewer #2: UKBiobank data were used to investigate seasonality of birth with mental health traits and brain morphology and microstructure measured from MRI. Depressive traits were associated with summer births as were significant increases in cortical thickness. Changes to diffusion measures were associated with winter births. The scientific reasoning behind this study described in the Introduction is that the season of birth affects brain structure and changes to brain structure are observed in mental health disorders.**

**The observed effects of seasonality of birth are weak and do not help clarify a confused literature and seasonality of birth effects include some areas that overlap with a recent meta-analysis of cortical thickness in cross-sectional primary literature of (diagnosed) MDD, although the direction is reversed, although the entire cohort is used in the linear model rather than focusing on any particular trait.**

**1. A more direct comparison of the underlying hypothesis would be that once seasonality of birth and prevalence is established for, as it would appear, MDD traits, then first undertake a case-control comparison of all participants with MDD traits and then a MDD traits summer compared to winter births. The expectation would be a pattern of differences similar to that seen in the literature with effect sizes increased in summer compared to winter.**
We thank the reviewer for this comment.

(a) A case-control comparison of all participants with MDD traits was conducted for all brain imaging measures and the methods for this have now been added to the manuscript (p12, lines 209-213) and below:

“First, models to examine associations between brain imaging measures and probable Major Depressive Disorder cases (P-MDD) were constructed. P-MDD included all participants who had been previously classified as P-RMDD or P-SEMDD cases, with P-MDD controls defined as those participants who were controls for both P-RMDD or P-SEMDD (See S2.5.1 for demographic table).”.

(b) This was supplemented by a case-control comparison for RMDD cases vs SEMDD cases for all brain imaging measures based on the differences we observed when they were examined separately. The methods for this analysis can also now be found in the manuscript (p12, lines 216-219) and below:

“Thirdly, additional models were constructed to investigate associations between brain imaging measures and P-RMDD cases compared to P-SEMDD cases (See S2.5.3 for demographic table). All covariates were kept consistent with the main analysis.”.

(c) Finally, the case/control comparison of summer born MDD cases compared to winter born MDD cases for all brain imaging measures was also conducted with methods reported in (p12, lines 213-216) and below:

“Secondly, models were constructed to explore associations between brain imaging measures and winter birth P-MDD cases compared to summer birth P-MDD cases. Seasonality was split into winter births (December, January, February) and summer births (June, July, August) and coded as a categorical variable (See S2.5.2 for demographic table).”.

**2. Alternatively, apply the linear model to predict brain imaging measures with a main effect of mental health trait and a trait x month interaction, expecting effects in brain regions that match to some extent with the literature. In short, a credible hypothesis is posited that should be directly addressed by the analysis strategy.**
We thank the reviewer for this comment. We also took on board this suggestion and have now supplemented the methods and results for this analysis in (p12-13, lines 219-225) and below. Please note, to ensure consistency with the main analysis, the “month interaction” was replaced with a “seasonality” interaction.

“Lastly, models were constructed to investigate associations between mental health traits and neuroimaging measures in the context of seasonality. For each neuroimaging measure a linear/fixed effect model was applied, retaining the same covariates as in the main analysis plus one mental health trait (n=4: P-RMDD, P-SEMDD, P-BD and P-UM) and an interaction term between the mental health trait and seasonality. As in the main analysis, mixed-effects models were used for bi-hemispheric measures. The same multiple correction strategy was applied as in the main analysis.”.

The results for the above analyses (both comments 1 and 2) can now also be found combined into pages 14-15 (lines 263-294) with multiple figures (Figures 3-4, S7.Fig and S8.Fig) also made available. Discussion of these analyses can be found on lines 302-307, 308-314, and 381-416. The abstract and conclusion section have also been lightly modified to reflect these new results. Full results for additional analyses are also available in the Supplementary materials (S2.5, S3.4-7, S4) with extended results in S5 and supplemental figures (S7 Fig and S8 Fig).
